# Medical Micro/Nanorobots in Precision Medicine

**DOI:** 10.1002/advs.202002203

**Published:** 2020-10-04

**Authors:** Fernando Soto, Jie Wang, Rajib Ahmed, Utkan Demirci

**Affiliations:** ^1^ Bio‐Acoustic MEMS in Medicine (BAMM) Laboratory Canary Center at Stanford for Cancer Early Detection Department of Radiology School of Medicine Stanford University Palo Alto CA 94304‐5427 USA; ^2^ Canary Center at Stanford for Cancer Early Detection Department of Radiology School of Medicine Stanford University Palo Alto CA 94304‐5427 USA

**Keywords:** diagnosis, medical imaging, micro/nanorobots, microsurgery, precision medicine, targeted delivery

## Abstract

Advances in medical robots promise to improve modern medicine and the quality of life. Miniaturization of these robotic platforms has led to numerous applications that leverages precision medicine. In this review, the current trends of medical micro and nanorobotics for therapy, surgery, diagnosis, and medical imaging are discussed. The use of micro and nanorobots in precision medicine still faces technical, regulatory, and market challenges for their widespread use in clinical settings. Nevertheless, recent translations from proof of concept to in vivo studies demonstrate their potential toward precision medicine.

## Graphical abstract

Miniaturization of medical robotic platforms has led to numerous applications that leverages precision medicine. In this review, the current trends of medical micro and nanorobotics for therapy, surgery, diagnosis, and medical imaging are discussed. Moreover, the technical, regulatory, and market challenges for their potential widespread use in clinical settings are addressed.

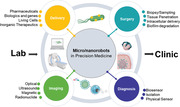

## 1 Introduction

**Figure 1:**
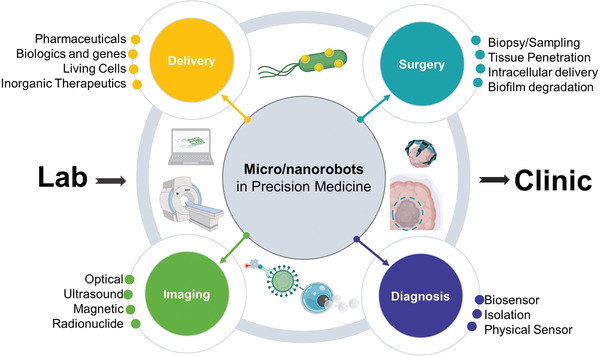
Schematic of the current trends of micro/nanorobotics in precision medicine, including delivery, surgery, diagnosis, and medical imaging applications.

The miniaturization of robotic platforms has the potential for advancing medical treatment and diagnosis of patients. These tiny robotic surgeons could give us access to remote and hard to reach sections of the body and perform diverse medical procedures^[^ ^1^, ^2^, ^3^, ^4^, ^5^ ^]^ Despite the progress of medical micro/nanorobots in the last decade, one of the unmet needs and significant challenges of this field relies on translating these tools toward widespread clinical use. In this direction, this review aims to illustrate recent trends in micro/nano robotic research, focusing on their use in precision medicine toward clinical transition (**Figure** **1**). In terms of this work, a medical micro/nanorobot is defined as an untethered micro/nanostructure that contains an engine capable of transforming diverse types of energy sources into mechanical force aimed toward performing a medical procedure.^[^ ^6^, ^7^, ^8^, ^9^, ^10^ ^]^ Although recent reviews have previously covered generalized or specific topics in the use of biomedical applications,^[^ ^11^, ^12^, ^13^, ^14^ ^]^ this review aims to bring together a comprehensive and thorough overview of the most recent developments of micro/nanorobots from a precision medicine perspective, highlighting the most promising research opportunities for the next decade that could have a profound impact on human health.

These areas include therapy, surgery, diagnosis, and medical imaging. Each of these areas aims to address different challenges in medicine. For example, motile micro/nanorobots could swim directly into target regions and deliver a precise dosage of a therapeutic payload. Thus, retaining their therapeutic efficacy while reducing side effects, which are a common problem when using a passive delivery approach with low localization efficacy. On the other hand, the use of micro/nanorobots for surgery could reach regions of the body not accessible by catheters or invasive surgery, allowing to sample tissue or deliver therapeutic payloads deep into diseased tissue. The use of tiny robotic surgeons could help reduce invasive surgical procedures, thus reducing patient discomfort and postoperation recovery time.

Micro/nanorobots have potential in medical diagnosis, by isolating pathogens or measuring physical proprieties of tissue in real‐time allowing to obtain a precise diagnosis of disease and vital signals. The integration of micro/nanorobots with medical imaging modalities would provide accurate positioning inside the body. These four topics have the potential to benefit human health by offering unique capabilities that their macro‐sized counterparts are not able to achieve. After a brief overview of the fundamentals of fabrication and powering of microrobots, each topic will be discussed thoroughly in Sections 2, 3, 4, 5, 6. Finally, we outline a roadmap identifying the main challenges and potential risks associated with the translation of medical microrobots from lab to clinic.

## 2 Fundamentals of Micro/Nanorobotics

Researchers in the micro/nanorobotic field commonly refer to the first generation of small‐scale robots as “micro/nanomotors” or “micro/nanoengines” defined as small scale structures capable of converting diverse energy sources into locomotion or actuation.^[^ ^15^, ^16^, ^17^, ^18^ ^]^ In contrast, a micro/nanorobot is a small scale structure capable of performing a preprogrammed task through mechanical actuation.^[^ ^6^ ^]^ Given that small scale robotic designs are quite different from their macroscale counterparts, it has been hard to define what should be considered as a micro/nanorobot. When a new research subfield emerges from a well‐established field, typically, its progress is judged from what it is not, rather than what it is.^[^ ^19^ ^]^ For example, when automobiles started to appear on the streets more than a century ago, they were referred to as “horseless carriages.”^[^ ^20^ ^]^ The design of small scale robots face distinct challenges when compared to internal combustion engines, electrical motors, or hydraulic and pneumatic devices. At microscopic scales, viscous forces dominate over inertial forces. Structures at such small scale have to consider environmental effects, such as Brownian motion, caused by random collisions of water molecules with microscale objects that interfere with the directionality of a motile micro/nanorobot.^[^ ^21^ ^]^

### 2.1 Fabrication of Microrobots

At small scales, locomotion is governed by low Reynolds numbers and Brownian motion, thus the primary consideration for designing micro/nanorobots relies on developing engines that are continuously “turn‐on” and generate enough force to overcome the drag forces from the environment.^[^ ^22^ ^]^ Therefore, the design and fabrication of small‐scale robots are driven by the need for active materials that can continuously convert diverse energy sources into locomotion. For example, chemically propelled microrobots require the asymmetric distribution of catalytic material to generate directional motion,^[^ ^23^ ^]^ magnetically propelled micromotors use magnetic materials to induce rotation of a microengineered structures^[^ ^24^ ^]^ and ultrasound propelled motors employ a structure with density asymmetries to generate pressure gradients that enable their locomotion.^[^ ^25^ ^]^ The first generation of micro/nanoengines for small scale robotics relied on simple fabrication procedures and geometries.^[^ ^26^ ^]^ These early nanorobots were fabricated by electrochemically reducing metallic correspondent salts inside nano/micro symmetrical pores.^[^ ^27^ ^]^ The advantage of this highly explored fabrication method is the large scale production (>100 0000 structures per batch), and the ability to intercalate different electroactive materials (metals, polymers, semiconductors) and designs (hollow tubes, porous wires) in the same construct.^[^ ^28^, ^29^, ^30^ ^]^ Another bottom‐up strategy is self‐assembly.^[^ ^31^ ^]^ This includes layer by layer assembly of sequentially charged materials,^[^ ^32^ ^]^ generating self‐organized polymers to create bowl shape stomatoyces filled in their interior with catalytic materials,^[^ ^33^ ^]^ and connecting colloids to make engineered structures^[^ ^34^ ^]^ and magnetic links.^[^ ^35^ ^]^

The use of thin‐film coatings over templates to generate asymmetric coated structures has also been explored for micro/nanoengine fabrication. For example, Janus micromotors were built by adding a catalytic thin film layer over half of a microsphere using e‐beam or sputtering deposition.^[^ ^36^, ^37^, ^38^ ^]^ On the other hand, atomic layer deposition‐based coatings (parylene, titanium oxide, silicon oxide) were used to cover most of the reactive surface except for a small opening, thus reducing the exposed reactive area.^[^ ^39^, ^40^ ^]^ The advantage of these methods is that there are many different types of commercially available microtemplates ranging from polymeric to metallic beads, with more recent examples of the use of biological and bioinspired templates.^[^ ^41^, ^42^, ^43^ ^]^

Other designs with more complex structures, such as microcoils or sophisticated geometries have been built using advanced techniques, including 3D printing,^[^ ^44^, ^45^, ^46^, ^47^, ^48^ ^]^ glancing angle deposition,^[^ ^49^, ^50^, ^51^, ^52^ ^]^ and rolled‐up lithography.^[^ ^53^, ^54^, ^55^ ^]^ These novel techniques offer new design capabilities allowing to add functionality by design. However, they are commonly more costly and have limited material selection.

Diverse methods are used to fabricate biohybrid micro/nanorobots.^[^ ^56^, ^57^ ^]^ For example, self‐assembly due to electrostatic interactions between living organisms and synthetic components is used to engineer biohybrid robots. Some microorganisms have either negative or positive charges. Thus, the surface charge of the synthetic component requires to be finely tuned to induce a long‐lasting interaction between the synthetic and biological entity. Recent publications have also demonstrated the ability to target binding of the synthetic component with either the head or the tail of a microorganism, based only on noncovalent interactions.^[^ ^58^ ^]^ Another strategy is based on the physical entrapment of functional nanostructures in the rough surface of a microorganism.^[^ ^59^ ^]^ Both strategies are attractive due to their simplicity. However, the lack of covalent interactions between the synthetic component and the microorganism surface makes it prone to detachment under environmental stress.

The covalent interaction via functionalization of the synthetic component surface with antibodies or chemical agents, which bind to the surface of the motile microorganism, has also been explored. This method ensures long‐lasting binding but limits the ability to release the synthetic component on demand.^[^ ^60^, ^61^ ^]^ Future developments of biohybrids could be assisted by the judicious selection and standardization of the motile microorganism. For example, various microfluidic device technologies containing a periodic array of pores,^[^ ^62^ ^]^ channels,^[^ ^63^, ^64^, ^65^, ^66^ ^]^ and pillars^[^ ^67^ ^]^ have been used to select and sort sperm with faster and specific morphology, where these can be used to make better micromotors^[^ ^68^ ^]^ In general, each fabrication method has unique advantages and challenges illustrated by the tradeoff of large‐scale production, material selection, resolution, and freedom of design. Future consideration for fabricating micro/nanorobots should take into consideration the safety of the materials in clinical environments and their fabrication scalability, as most micro/nanorobot research is tailored for laboratory settings.

### 2.2 Robotics Engines at Small Scales

**Figure 2:**
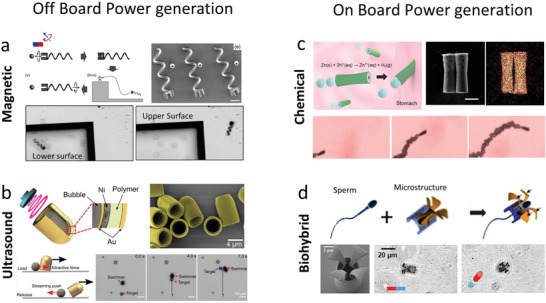
Powering mechanism for micro/nanorobots. a) Magnetically propelled microrobot based on rotating microcoil. Reproduced with permission.^[^ ^82^ ^]^ Copyright 2012, Wiley. b) Ultrasound propelled microrobot powered by cavitating microbubble. Reproduced with permission.^[^ ^94^ ^]^ Copyright 2019, AAAS. c) Chemically propelled motor based on zinc microtube, the microrobot converts gastric fluid into gas bubbles that generate propulsion trust. Reproduced with permission.^[^ ^111^ ^]^ Copyright 2015, American Chemical Society. d) Biohybrid microrobot based on the integration of a sperm with a synthetic structure. Reproduced with permission.^[^ ^129^ ^]^ Copyright 2018, American Chemical Society.

Nature has developed diverse mechanisms to achieve motion at small scales. Many microorganisms possess chemical rotors that enable them to power flagella or cilia, actuated to produce a corkscrew or beading motion leading into locomotion.^[^ ^69^, ^70^, ^71^ ^]^ Such propulsion mechanism has been the inspiration for rotating synthetic microrobots,^[^ ^72^, ^73^, ^74^ ^]^ where synthetic helical microstructures,^[^ ^75^, ^76^, ^77^ ^]^ flexible filaments,^[^ ^78^, ^79^ ^]^ or tumblers^[^ ^80^, ^81^ ^]^ rotate in axis to that of a bacterial flagellum. Each individual microrobot is energetically independent of the other microrobots rather than been dragged toward the direction given by the magnetic field (**Figure** **2**a).^[^ ^82^, ^83^, ^84^ ^]^

Ultrasound is another external energy input to power microrobots.^[^ ^85^, ^86^, ^87^, ^88^ ^]^ The first ultrasound powered micro/nanorobots consisted of metallic asymmetric nanowires powered by a standing wave, in which the generation of localized acoustic streaming stress over the asymmetric nanowire surface produced the driving force for motion.^[^ ^89^, ^90^ ^]^ More recent developments have utilized high intensity focused ultrasound to induce the rapid vaporization of chemical fuels that result in bullet‐like motion microrobots^[^ ^91^ ^]^ and the use of a traveling wave to induce the oscillation of a bubble trapped within a microrobots structure (Figure 2b).^[^ ^92^, ^93^, ^94^ ^]^

Chemical gradients used to power the bacterial flagella have been a source of inspiration for chemically propelled motors. These micromotors use local energy conversion of fuels from the environment into motion.^[^ ^95^, ^96^, ^97^, ^98^ ^]^ The propulsion mechanisms included self‐electrophoresis,^[^ ^99^, ^100^, ^101^ ^]^ where an asymmetric fuel decomposition sustains the propulsion of the nanorobot, or bubble propulsion generated by the ejection of gas microbubbles continuously formed inside the catalytic sites of the microrobot engine.^[^ ^102^, ^103^ ^]^ The first generation of chemically powered micromotors used platinum surfaces as the catalytic engine and peroxide as the fuel. Nevertheless, new fuels and propellant materials have emerged as a biocompatible alternative. For example, enzymes have substituted platinum, allowing them to use a variety of biomolecules, such as glucose or urea as fuel.^[^ ^104^, ^105^, ^106^, ^107^, ^108^, ^109^, ^110^ ^]^ Moreover, biodegradable metals, such as zinc or magnesium have been used as propellants. These can react with the acidic environment of the stomach (Figure 2c).^[^ ^111^ ^]^ The later materials are of particular interest because the metallic propellant is degraded after use, leaving nontoxic byproducts behind.^[^ ^112^ ^]^ Optical^[^ ^113^, ^114^, ^115^, ^116^, ^117^, ^118^, ^119^ ^]^ and electrical fields^[^ ^120^, ^121^, ^122^, ^123^, ^124^, ^125^ ^]^ have also been used to enhance the reaction rate of catalytic materials to produce locomotion, although they are less commonly used in biomedical applications.

Biohybrid robots are built by coupling motile microorganisms (responsible for locomotion), with synthetic structures (to provide additional functionalities) (Figure 2d).^[^ ^126^, ^127^, ^128^, ^129^, ^130^, ^131^ ^]^ More recently, advances in synthetic biology have made possible the ability to enhance the capabilities of the motile microorganism without the use of artificial components. For example, genetically engineered bacteria have been programmed to generate diverse active components, such as magnetic particles,^[^ ^132^, ^133^ ^]^ gas‐filled microstructures,^[^ ^134^, ^135^ ^]^ therapeutic payloads,^[^ ^136^ ^]^ or responsive probes.^[^ ^137^ ^]^

In a nutshell, locally powered microrobots have built‐in energy conversion on their active surfaces or exploited the autonomous motility of microorganisms. On the other hand, externally powered microrobots are self‐propelled as a result of the interaction between an external field, the robot structure and the media in which they move. Therefore, each powering modality system has a unique advantage. Locally powered micromotors are ideal where autonomy is desired, such as microscale mixing, environmental remediation, and low precision drug delivery. Nevertheless, external power sources are ideal for applications, such as microsurgery or targeted delivery, where control is essential.

## 3 Targeted Delivery

The ability to guide micro/nanorobots directly into diseased tissue could serve as a dynamic platform for the delivery of diverse types of cargoes. For example, pharmaceutical, biologics, living cells, and inorganic therapeutics. Moreover, the stimuli that propels and guides micromotors can be used to improve drug targeting by inducing trigger the release of the therapeutic payload when the micromotor reaches a specific location.^[^ ^138^, ^139^, ^140^, ^141^ ^]^ Micro/nanorobots use diverse methods to carry therapeutic agents, including the use electrostatic or covalent interactions to entrapping them directly on their surface, or by embedding them inside responsive materials. The subsequent release of the therapeutic cargoes is based on diverse mechanisms, including autonomous release induced by a change in environment (changes in pH or temperature) and the use of triggered release induced by application of external fields (near infrared, ultrasound field). In both cases regardless of the loading methodology, either local or external stimuli can result in the change of surface proprieties or degradation of a materials that entails the release of the loaded therapeutic cargo.

### 3.1 Pharmaceutical Drugs

Pharmaceutical drugs consist mostly of small synthetic chemicals designed to treat and prevent diseases. Regardless of the type of administration, the efficacy of drug formulations is often compromised by poor pharmacokinetic proprieties of pharmaceutical drugs, such as short half‐life, limited biodistribution, and rapid clearance from the body. Therefore, repeated administrations in high dosages are inevitable to induce the desired therapeutic effect, which could lead to increased toxicity and side effects (e.g., cardiotoxicity).^[^ ^142^, ^143^ ^]^ In this direction, micro/nanorobots have the potential to overcome this challenge by offering a motile platform capable of delivering a precise dosage in the target area rather than relying on the systemic release of large therapeutic dosages.^[^ ^144^, ^145^, ^146^, ^147^, ^148^ ^]^

One of the first examples of micro/nanorobots for delivery of pharmaceuticals was reported a decade ago, where a catalytic nanowire nanorobot was used as a nanoshuttle to pick up, transport, and release doxorubicin/iron oxide loaded poly D,L‐lactic‐co‐glycolic acid (PLGA) liposomes. The microrobot contained a nickel segment that served as both a magnetic navigation guide and anchor for PLGA liposomes through weak magnetic interaction. The rapid change of direction resulted in the dislodging of the PLGA liposome due to the increased drag force imposed on the particle.^[^ ^149^ ^]^ A similar approach was reported using a flexible magnetic microrobot composed of a nickel head and a flexible silver tail.^[^ ^150^ ^]^ The use of nickel/titanium helical microrobots was reported to load calcein loaded liposomes. The vesicles were adsorbed over the TiO_2_ surfaces of the microrobot via electrostatic interaction.^[^ ^151^ ^]^ Superparamagnetic microengines arranged in a train‐like structure have also been used to capture cells and simultaneously release doxorubicin by diffusion. In this work, the micro/nanorobot was functionalized with tosyl groups on its outer surface, which served to bind cancer cells through their surface and load drugs such as doxorubicin.^[^ ^152^ ^]^

**Figure 3:**
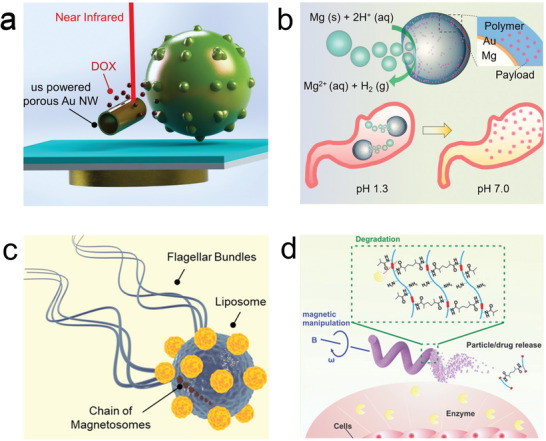
Micro/nanorobot based delivery of pharmaceuticals. a) Ultrasound propelled nanowires for near‐IR triggered delivery of doxorubicin. Reproduced with permission.^[^ ^156^ ^]^ Copyright 2014, Wiley. b) Magnesium powered microrobot for stomach pH neutralization and sustain drug release. Reproduced with permission.^[^ ^172^ ^]^ Copyright 2017, Wiley. c) Biohybrid micromotor consisting of a magneto‐tactic bacterium transporting liposomes. Reproduced with permission.^[^ ^190^ ^]^ Copyright 2016, Springer Nature. d) Magnetically powered microrotor with enzymatic biodegradation for triggered drug release. Reproduced with permission.^[^ ^197^ ^]^ Copyright 2018, Wiley.

Pharmaceutical agents have also been entrapped directly on the surface of micro/nanorobots by using electrostatic interactions. The use of electrostatic forces was reported to load the positively charged brilliant green antiseptic drug into a negatively charged polypyrrole–polystyrene sulfonate segment of an ultrasound propelled nanorobot. The electrostatic interaction was stable at pH 7. On the other hand, when the environmental pH became relatively acidic (pH 4) the polypyrrole–polystyrene material segment was protonated, resulting in a triggered release of the loaded Brilliant green drug molecule.^[^ ^153^ ^]^ In another example, reduced graphene oxide/platinum microrockets were used to transport doxorubicin. The reduced graphene oxide served to load the pharmaceutical drug via *π*–*π* interactions. This method presented a unique trigger‐release mechanism based on electrochemical stimuli that disrupt the interactions between the doxorubicin and the graphene surface of the micro/nanomotor.^[^ ^154^ ^]^ Moreover, the use of an electrochemical stimuli as a release mechanism was further expanded by using bismuth coatings to load the therapeutic payload. The injection of electrons into the motor surface caused electrostatic repulsions and the loading of doxorubicin.^[^ ^155^ ^]^ Ultrasound propelled porous nanowire surface was functionalized with an anionic coating that permitted the electrostatic loading of doxorubicin into the micro/nanorobot structure. The porous segment was responsible for increasing the drug loading capacity and for facilitating the release by photothermal effect upon radiation of near‐infrared light^[^ ^156^ ^]^ (**Figure** **3**a). Similarly, mesoporous chemically propelled Janus microrobots were used for near‐infrared triggered delivery.^[^ ^157^ ^]^ The use of pH‐sensitive polymers is potentially ideal for autonomous and trigger‐release of pharmaceutical drugs next to cancer sites, as the byproducts of cancer cell metabolites result in a local acidic environment.^[^ ^158^, ^159^, ^160^ ^]^ Another concept for drug delivery relies on inducing drug release based on mechanical rotation.^[^ ^161^, ^162^ ^]^ A microrobot powered by rotating and alternating currents was used to deliver Nile blue loaded onto the nanorobot via weak electrostatic interactions. The release of this model drug was controlled by modulating the mechanical rotation rate of the nanorobot.^[^ ^163^ ^]^ Moreover, the use of urea powered nanorobots were reported to enhance doxorubicin release kinetics. A mesoporous silica shell was loaded via electrostatic interactions with a pharmaceutical, doxorubicin, and urease, a biocatalytic enzyme capable of decomposing urea and harnessing the chemical energy into fluid mixing.^[^ ^164^ ^]^ The limitation of electrostatic loading is that multiple environmental factors can result in dislodging of the loaded pharmaceutical due to the weak binding of the therapeutic cargo with the micro/nanorobot surface.

To increase drug release selectivity, the pharmaceutical compounds have also been directly embedded inside responsive materials to achieve controlled release.^[^ ^165^ ^]^ For example, microrockets were built via layer‐by‐layer. The assembly consisted of sequential layers of positively charged chitosan and negatively charged sodium alginate, where the therapeutic payload, doxorubicin, was internalized. The microrockets were directed and attached next to HeLa cells and subjected to an ultrasound pulse that locally released the doxorubicin to the cell inducing signs of apoptosis.^[^ ^166^ ^]^ The same group also reported the use of gelatin‐based microrockets^[^ ^167^ ^]^ and catalase powered layer‐by‐layer Janus motors,^[^ ^168^ ^]^ for light‐triggered release using near‐infrared of doxorubicin from the bio‐gel matrix. Moreover, soft nanorobots were fabricated via self‐assembled block copolymers loaded with platinum nanoparticles and doxorubicin. The bilayer structure of the nanomotor could load both hydrophilic and hydrophobic pharmaceuticals.^[^ ^169^ ^]^ In their follow‐up study, they developed a triggered chemical response to glutathione, which cleaved the polyethylene glycol shell, thus releasing the therapeutic payload.^[^ ^170^ ^]^

The use of magnesium powered microengines has been used toward targeted drug delivery inside the gastrointestinal tract, using biofluids as fuel. Magnesium microengines coated with a poly(N‐isopropylacrylamide) have been used to induce temperature‐controlled delivery of fluorescein isothiocyanate as a model drug in simulated body fluids or blood plasma.^[^ ^171^ ^]^ The use of acid‐driven micromotors was reported based on Mg coated with a cargo‐containing pH‐responsive polymer, to neutralize gastric acid in a mouse model. The consumption of the magnesium core by the acidic environment in the gut led to the depletion of hydrogen protons from the gastric environment, thus raising the pH to a neutral environment without the requirement of a proton pump inhibitor. The increase in pH resulted in the degradation of the pH sensitive polymer containing the loaded drug model (Figure 3b).^[^ ^172^ ^]^ Taking advantage of this principle, further work demonstrated the first in vivo micro/nanorobot therapeutic application by delivering clarithromycin, loaded into a PLGA layer over the magnesium propellant, as a model antibiotic to treat *Helicobacter pylori* infection inside a mouse stomach.^[^ ^173^ ^]^ More recently, magnesium microrobots loaded with ampicillin were reported for bacterial treatment.^[^ ^174^ ^]^ Another animal study used a zinc/iron engine toward gastrointestinal delivery tool of doxorubicin.^[^ ^175^ ^]^ The use of magnesium‐based micromotor was reported to enhance therapeutic efficacy doxorubicin by a synergistic effect on site hydrogen generation.^[^ ^176^ ^]^ These tiny tools have been integrated into pills^[^ ^177^ ^]^ and multicompartmentalized segments for delivery with delay actuation release for improving their loading and stability.^[^ ^178^, ^179^ ^]^

Biotemplated chemically propelled micro/nanorobots have also been used as carriers for different pharmaceuticals. Platinum‐coated plant virus capsules, including brome mosaic virus and cowpea chlorotic mottle virus, were used as nanotemplates for the delivery of tamoxifen, loaded inside their hollow interior. When the virus was internalized inside a cell, its structure denaturalized due to a lower pH environment, which resulted in the autonomous release of the drug.^[^ ^180^ ^]^ Another type of biotemplate was fabricated by turning red blood cells into microrobots.^[^ ^181^, ^182^ ^]^ This biorobot was fabricated by incorporating quantum dots, doxorubicin, and magnetic nanoparticles into red blood cell micromotors. The fluorescent emission of both quantum dots and doxorubicin provided direct visualization of their loading inside the red blood cell motors at two distinct wavelengths. They demonstrated that these red blood cell micromotors could transport imaging and therapeutic agents at high speed and spatial precision through a complex microchannel network.^[^ ^183^ ^]^ Pollen structures were used to obtain different microengine proprieties by taking advantage of their resilient outer layer and hollow interior.^[^ ^184^, ^185^ ^]^ More recently, a fully organic microrobot consisting of urease‐powered Janus platelet micromotor was engineered by immobilizing enzyme onto the surface of natural platelet cells toward selective adhesion to cancer cells and subsequent delivery of doxorubicin.^[^ ^186^ ^]^ Microorganism‐driven micro/nanorobots have also been used as targeted drug delivery systems. Motile sperm loaded with doxorubicin were integrated into a 3D‐printed magnetic tubular microstructure that permitted magnetic guidance and assistance to push against the tumor site.^[^ ^129^ ^]^

The use of electrostatic self‐assembly was used to fabricate biohybrid sperms^[^ ^187^ ^]^ and Chlamydomonas reinhardtii based microrobots carrying magnetic nanoparticles and therapeutic loads.^[^ ^188^ ^]^ In another example, *Escherichia coli* (*E. coli*) was captured with drug‐loaded polyelectrolyte multilayer microparticles containing magnetic nanoparticles, and doxorubicin. This work reported in vitro magnetic guided delivery of doxorubicin encapsulated in the multilayer microparticle toward 4T1 breast cancer cells.^[^ ^189^ ^]^ The use of magnetotactic bacteria was used to carry drug‐loaded nanoliposomes (Figure 3c).^[^ ^190^ ^]^ An external magnetic field was used to guide the biohybrids toward cancer cells.^[^ ^191^ ^]^ Another advantage of the use of biohybrids as motile carriers relies on their build‐in sensing capabilities because microorganisms can detect chemical cues in their environment to find food or avoid danger. Thus, hybrid neutrophil micromotors were loaded with therapeutic cargoes. Neutrophils can detect chemoattractant gradients produced in inflammatory sites and move eliminating pathogens.^[^ ^192^ ^]^ Doxorubicin‐loaded biohybrid sperm have also shown a chemotactic movement to a “sperm activating and attracting factor” molecule. The biohybrid platform was tested in vivo for the targeted delivery of doxorubicin into human ovarian cancer cells.^[^ ^59^ ^]^ These contributions are of great interest as they use biodegradable materials to transport the therapeutic payloads, leaving nontoxic byproducts behind.

Untethered mobile microrobots have leveraged minimally invasive theragnostic functions precisely and efficiently in hard‐to‐reach, confined, and delicate inner body sites.^[^ ^193^ ^]^ The use of 3D printed magnetically powered soft biodegradable micro/nanorobot structures with near‐infrared triggered tunable doxorubicin delivery were reported. The microstructure was composed of chitosan functionalized photocleavable linkers to load doxorubicin. The tunable application of a near‐infrared external field was used to modulate doxorubicin release kinetics. Moreover, lysozyme, a natural enzyme found in the human body, was used to degrade the microrobot structure without producing cytotoxic degradation products.^[^ ^194^ ^]^ An expansion of this work demonstrated that a gelatin methacryloyl micro/nanorobot could be used to deliver drugs based on polymer swelling.^[^ ^195^ ^]^ Rolling micro/nanorobots were reported to swim against the bloodstream, and being able to detect target cancer cells using cell‐specific antibodies, and induce near infrared triggered release of a doxorubicin payload.^[^ ^196^ ^]^ The safety tests conducted in the 3D printed GelMa microrobots indicated the limited cytotoxicity after enzymatic degradation (Figure 3d).^[^ ^197^ ^]^

The use of metal–organic frameworks (MOF) have been also used for smart therapeutic release based on pH changes. A zeolitic imidazole framework‐8 coated microhelix was able to release model drugs based on changes in the local pH environment.^[^ ^198^ ^]^ Microrobots composed of multiwalled carbon nanotube coated with Fe_3_O_4_ nanoparticles and antiepithelial cell adhesion molecule antibodies were used to target and deliver doxorubicin toward spheroid tumors.^[^ ^199^ ^]^ Magnetically powered soft robots used the piezoelectric effect to trigger an electrostatic release of doxorubicin.^[^ ^200^ ^]^ A flexible mesoporous silica nanotube coated with CoFe_2_O_4_ magnetic nanoparticles was used as a motile drug carrier. The pores of the structure were covered by G‐quadruplexes, consisting of highly ordered DNA structures. These served as valves that opened under magnetic actuation by a conformal change of a rigid G‐quadruplex structure and a random single‐stranded actuation. Thus, the valve opening released the 6‐carboxyfluorescein, model drug.^[^ ^201^ ^]^

### 3.2 Biologics

Another relevant area of research has been focused on the micro/nanorobot delivery of biological components, such as proteins, tissue plasminogen activator for thrombolysis, viral vaccines, or antibodies. In contrast to synthetic pharmaceutical drugs, biologics are therapeutic agents that are commonly produced by living systems, including proteins or small segments of a biological component.^[^ ^202^, ^203^ ^]^ These therapeutics aim to use compounds already produced inside the body as an active agent.

**Figure 4:**
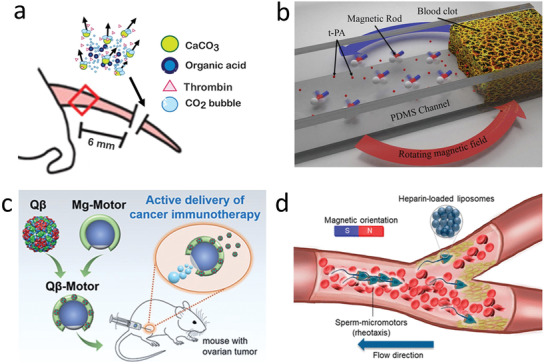
Micro/nanorobot based delivery of biologics and genes. a) CaCO_3_ powered microrobot for the enhanced delivery of thrombin through flowing blood and halt hemorrhage. Reproduced with permission.^[^ ^206^ ^]^ Copyright 2015, AAAS. b) Accelerated catalytic reactivity of tissue plasminogen activator (t‐PA) mediated blood clot degradation by magnetically powered microrobots. Reproduced with permission.^[^ ^211^ ^]^ Copyright 2014, American Chemical Society. c) Use of magnesium powered microengines to deliver virus vaccines in a mouse tumor model. Reproduced with permission.^[^ ^216^ ^]^ Copyright 2020, Wiley. d) Sperm driven microrobot for heparin delivery capable of going against the flow. Reproduced with permission.^[^ ^219^ ^]^ Copyright 2020, American Chemical Society.

For example, electrically powered rotor nanorobots were used to deliver tumor‐necrosis factor. Gold nanowires were functionalized with a hydrophobic layer of 1‐dodecanethiol, enabling absorption of tumor‐necrosis factor on the surface of the nanorobot. This work demonstrated that a single nanorobot could carry and deliver a threshold of tumor necrosis factor to stimulate the activation of canonical nuclear factor‐kappaB transcription factor inside a single cell, simulating immune chain reaction signaling.^[^ ^204^ ^]^ In another work, ultrasound propelled nanowires used pH‐responsive glucose oxidase/phenylboronic acid supramolecular nanovalves to release insulin from a mesoporous silica segment. This approach had a gated responsive release, as insulin only was released in the presence of glucose. The glucose oxidase enzyme catalyzed glucose into gluconic acid, which decreased the local pH and induced protonation of the phenylboronic acid groups opening of the insulin‐loaded reservoirs located at the silica segment.^[^ ^205^ ^]^ Chemically propelled microrobots were used to deliver thrombin (coagulant) upstream through the blood vessels toward halt hemorrhage in pig and mouse animal models.^[^ ^206^, ^207^ ^]^ The engine of the micro/nanorobot used the chemical degradation of carbonate and tranexamic acid, to generated gas bubbles as a propulsion source (**Figure** **4**a).

The recombinant tissue plasminogen activator (rtPA or tPA), a protein used to break the cross‐linked fibrins that provide blood clot structure, has been a highly studied target for micro/nanomotors in the precision medicine tools for delivery. While t‐PA is an Food and Drug Administration (FDA) approved biological, there is an important risk of side effects, such as symptomatic intracranial hemorrhages.^[^ ^208^ ^]^ Therefore, this protein requires control of the dosage as well as effective delivery to the blood clot to reduce secondary risks associated with stroke treatment. The first targeted delivery methods for this biological consisted of micro and nanocarriers transporting a small dose of tPA with magnetic nanoparticles coated on a biodegradable polymeric matrix driven to the blood clot via external magnetic fields.^[^ ^209^ ^]^ Taking advantage of the shear stress caused by the constriction in a vessel, a passive diffusion strategy for tPA delivery was developed. Coated tPA‐based microaggregates of PLGA nanoparticles, similar in size to natural platelets, were naturally targeted to the blood clot where tPA induced rapid dissolution of the obstruction and allowed normal flow dynamics.^[^ ^210^ ^]^An active strategy using rotating nickel‐based magnetic nanorobots to improve the local mass transport of t‐PA at the blood clot interface has also been reported. The robots were used along with free t‐PA to act as an independent input for efficiency. Using a polydimethylsiloxane fluidic channel model to mimic blood vessels, they directed the motor to the clot, produced active motion of the nanorobot, and enhanced the thrombolysis by hydrodynamic convection (Figure 4b).^[^ ^211^ ^]^ The clinical application of these nickel‐based robots was limited because of the toxicity of the material. Therefore, other groups attempted using biocompatible magnetic micromotors, based on superparamagnetic iron oxide (Fe_3_O_4_). The tPA was covalently loaded onto the Fe_3_O_4_ nanomaterials. Iron oxide nanobots loaded with tPA targeted clots in the brain under magnetic guidance. The application of a rotational magnetic field allowed to perform enough mechanical force to perforate the clot and release the tPA within 30 min into the clot. This approach enabled the plasminogen to reach new binding sites and enhanced the susceptibility of the clots to lysis.^[^ ^212^ ^]^ However, a limitation of these ferromagnetic Fe_3_O_4_ nanorods t is the tendency of these structures to aggregate. New works have tested the capabilities of porous superparamagnetic Fe_3_O_4_—C nanorobots that encapsulate tPA to deliver this biological in a target area. After the injection of a solution containing the robots near the brain, they target the blood clot occluded in the middle cerebral artery in the mice. The microrobots were guided by an external magnet, located in proximity to the blood clot. In this case, the clot was dissolved via both tPA (chemical lysis) and rotating nanorobots (mechanical lysis) with the aid of an external rotating magnetic field. More importantly, the microcarriers did not cause liver or kidney damage and could be discharged from the kidney and collected in urine with a magnet or from the biliary system since they were found in bile tracts.^[^ ^213^ ^]^ More recently, the use of microgel spherical microrobots embedded with aligned magnetic nanoparticles toward thrombolysis by tPA were reported.^[^ ^214^ ^]^

Vaccines have been another biological preparation that have been explored for target delivery using micro/nanorobots. To maximize the efficiency of oral vaccines, biomimetic self‐propelling microrobots have been reported to deliver an attenuated vaccine in mice. In a recent paper, they deliver Staphylococcal *α*‐toxin, a hemolytic factor secreted by *Staphylococcus aureus*. By using magnesium‐based microrobots, the vaccine was delivered to the target zone. These microspheres were coated with three more layers: a toxin‐inserted red blood cell membrane, chitosan, and pH‐responsive enteric polymer layers. The first one was used to detain and neutralize a toxic antigenic payload; the second acted as a mucoadhesive for promoting intestinal localization and the third protected oral drugs from the harsh acidic conditions of the stomach.^[^ ^215^ ^]^ Virus‐like nanoparticle bacteriophage Q*β* has also been coated on biocompatible Mg‐motors for cancer immunotherapy applications in mice (Figure 4c). They were used for peritoneal ovarian tumor treatment. Active delivery in the peritoneal space of ovarian tumors was achieved, and the local distribution and retention of Q*β* virus improved versus passive treatment.^[^ ^216^ ^]^ Despite challenges, such as the potential for generating systemic immunity that is still required to overcome using this vaccine, these platforms show the first steps of micro/nanorobots in the delivery of biologicals in vivo and great promise in clinical scenarios.

Micro/nanorobots have also been implemented in medical tools, such as microneedles to enhance payload delivery. Microneedles have been clinically used to deliver drugs on the epidermis because of its painless effect and localized delivery of target drugs. However, their therapeutic payload distribution is limited by passive diffusion. Recently, magnesium particles have been included as an example of microrobots in degradable microneedle platforms to deliver anti‐CTLA‐4 antibodies autonomously and actively in a dermal melanoma model in mice. The magnesium engine of the microrobot reacted with the interstitial fluid in the epidermis, leading to a rapid hydrogen production, which generated enough force to open dermal barriers and produce a rapid antibody delivery.^[^ ^217^ ^]^ In vivo studies using near infrared powered nanorobot reported an increased efficacy urokinase toward thrombus therapy, when compared to passive delivery.^[^ ^218^ ^]^ Chemically triggered microrobots do not require external stimuli to improve the therapeutic outcome. Therefore, they do not need expensive and bulky external systems commonly used for triggering other active delivery, such as occurring with previously described magnetic micromotors. Sperm driven synthetic horn‐like microstructure loaded with heparin, a naturally occurring protein with an anticoagulant function, demonstrated efficient swimming against blood flow (Figure 4d). The biohybrid robot, collectively assembled, achieved efficient locomotion due to the reduction of the drag forces from the fluid. This approach offers unique opportunities to transport anticoagulant upstream too hard to reach regions due to heavy bleeding. Furthermore, sperm have unique potential as an engine of micro/nanorobots because of its limited proliferation or secretion of toxic byproducts and the inhibition of the immune reaction due to its protein‐rich surface.^[^ ^219^, ^220^ ^]^

### 3.3 Living Cells

**Figure 5:**
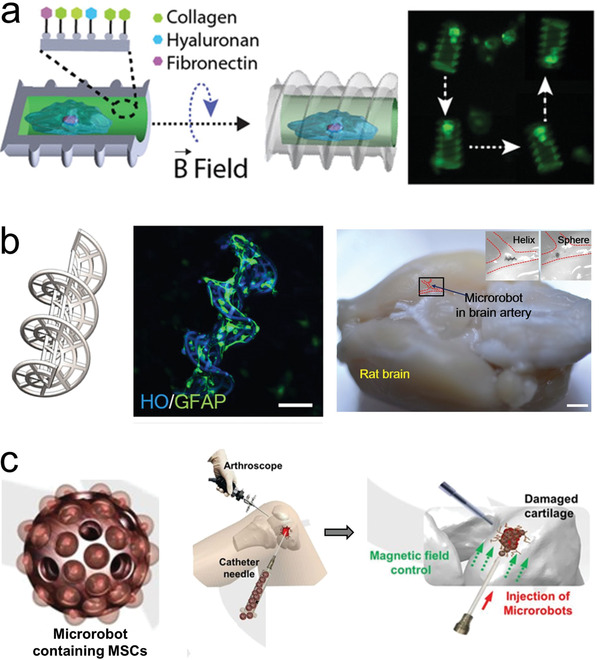
Use of microrobots as a scaffold for cell transport. a) Microrobot carrier with programable surfaces to control cell differentiation. Reproduced with permission.^[^ ^223^ ^]^ Copyright 2019, Wiley. b) Neuron loaded microhelix robot for brain delivery. Reproduced with permission.^[^ ^230^ ^]^ Copyright 2019, AAAS. c) Adipose‐derived tissue loaded microrobot for knee cartilage regeneration. Reproduced with permission.^[^ ^231^ ^]^ Copyright 2020, AAAS.

Recent developments in the use of micro/nanorobots as cell carriers offer a unique opportunity for regenerative medicine. The ability to deliver cells directly into the target tissue or stem cell niche could increase their retention rate and survival. Additionally, it could help address some of the significant challenges of regenerative cell transplantation.^[^ ^221^ ^]^ Taking advantage of the large loading capacity of micro/nanorobots, they can be engineered with various types of cells and possess different biological features. One strategy consists of using their microrobot surface as a cell culture scaffold, serving as a mechanical support for the cells to grow over a motile structure. SU‐8 microcages, coated with nickel for magnetic response and titanium for compatibility, were used to grow human embryonic kidney 293 cells and subsequently controlled by external magnetic fields.^[^ ^222^ ^]^ Microrobotic cell scaffolds made of programable materials were used for inducing biochemical and biophysical cues that regulated cell fate before and during their targeted delivery. The motile scaffold consisted of a hollow magnetic cylinder wrapped by a double helix. The inner cavity walls comprised of diverse niche components, including collagen, hyaluronan heparin, and fibronectin, entrapped in a gelatin matrix. This inner wall served to increase the adhesive stability of a transported cell, protect it from undergoing differentiation during transport, and directing it toward the desired lineage. The stem cell loaded microrobot could move along predetermined trajectories under external rotating magnetic fields (**Figure** **5**a). Moreover, the microrobot scaffold demonstrated the ability to induce preosteogenic differentiation of transported stem cells by including bone morphogenetic protein‐2 embedded inside the inner matrix.^[^ ^223^ ^]^ Larger functionalized hydrogel‐based robots have been used as cell carriers with the advantage of generating assemblies of different types of cells.^[^ ^224^, ^225^ ^]^ Our group has also expanded this concept by developing ultrasound‐based fabrication of highly packed cells embedded in hydrogel ring shape microstructures, which present a promising future for developing fully degradable microrobot scaffolds for the transport of multiple cells into a single structure.^[^ ^226^ ^]^

In another case, biodegradable microrobot scaffolds fabricated by 3d printing, were used toward targeted neuronal cell delivery for neurodegenerative disease therapy. The microrobot hydrogel chassis served as a scaffold to support neuronal cell growth and was enzymatically degraded after finishing the cell delivery. The microrobots were embedded with magnetoelectric nanoparticles, composed of a CoFe_2_O_4_ core and a BiFeO_3_ shell. The magnetoelectric nanoparticles served to induce motile magnetic actuation under low magnitude rotating magnetic fields. Moreover, the nanoparticles generated a magnetostrictive transient charged surface that was used to stimulate the differentiation of SH‐SY5Y, a neuroblastoma cell line.^[^ ^227^ ^]^ Degradable helical microrobots fabricated using a microfluidic chip also demonstrated the scalable fabrication of NIH 3T3 loaded structures that could stack forming larger cell‐loaded aggregates.^[^ ^228^ ^]^

Microrobots have already been tested using in vivo models to evaluate target stem cell delivery. Burr‐like magnetically propelled spherical microrobots were used for carrying and delivering cells in different animal models, including transport of MC3T3‐E1 cells into the yolk of a zebrafish embryo and the delivery of GFP in HeLa cells located inside the left dorsum of the nude mice. In the latter case, the fluorescence intensity increased after 4 weeks, indicating the successful proliferation of the delivered cells.^[^ ^229^ ^]^ Microrobots delivered hippocampal neural stem cells capable of inducing their proliferation and differentiation into astrocytes, oligodendrocytes, and neurons. They demonstrated that the micromotors could transport colorectal carcinoma cancer cells to tumor microtissue in a liver‐tumor micro organ‐on‐chip in vitro. The nanorobots could also navigate through the mesenchymal stem cells in a nude mouse brain intraperitoneal cavity and rat brain blood vessel in vivo (Figure 5b).^[^ ^230^ ^]^

Microrobots were also used for human adipose‐derived mesenchymal stem cells for knee cartilage regeneration using an in vivo rabbit model (Figure 5c). The microrobot scaffold consisted of a spherical PLGA microstructure decorated with ferumoxytol magnetic nanoparticles. After mesenchymal stem cells were loaded and grew over the surface of the microrobot, they were injected in the wounded knee of the rabbit. The application of an oscillating magnetic field served to enhance the motion of the microrobot to fill and accumulate at the target site (defects in the knee). Once in there, a permanent magnet was used to hold in place the cell loaded microrobots facilitating the adhesion and proliferation of the stem cells in the target location.^[^ ^231^ ^]^

**Figure 6:**
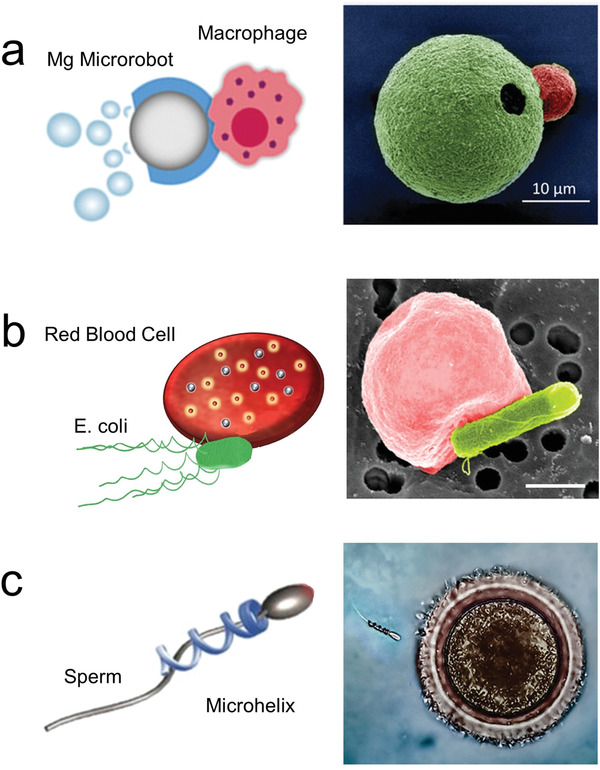
Microrobot as carrier of individual cells. a) Chemically powered microrobot transporting macrophage attached via electrostatic interactions. Reproduced with permission.^[^ ^232^ ^]^ Copyright 2019, Wiley. b) Biohybrid microrobot transporting red blood cell. Reproduced with permission.^[^ ^233^ ^]^ Copyright 2018, AAAS. c) Magnetically powered microhelix transporting sperm cell. Reproduced with permission.^[^ ^235^ ^]^ Copyright 2016, American Chemical Society.

Microrobots have also been used to transport individual cells without functioning as a scaffold, but rather by using chemical interactions or physical stimulation. Different types of blood cells have been coupled with motile robots to take advantage of their biological function. For example, magnesium‐based biohybrid micromotors system were integrated with live macrophage cells. This system included the biocompatible propulsion of the magnesium core engine from the microrobot and the biological functions of the microphage, which produced endotoxin neutralization (**Figure** **6**a).^[^ ^232^ ^]^ *E. coli* biohybrid microrobots were used to transport a live red blood cell (erythrocyte) via a biotin‐avidin‐biotin functionalization (Figure 6b). As both sections of the biohybrid robot were “soft,” they could maintain the interaction, after passing through a microfluidic channel smaller than their size.^[^ ^233^ ^]^ In this case, the red blood cell was used as a carrier, although it has the potential to be used as a sponge to capture toxins from blood samples.^[^ ^234^ ^]^

Magnetically powered microrobots have been proposed as carries of sperm. The microrobot consisted of microhelice structures that can capture and transport sperm cells inside their hollow interior (Figure 6c).^[^ ^235^, ^236^ ^]^ Micro/nanorobots have been also used to manipulate and transport oocytes/zygotes. For instance, microrobotic arms reported the ability to surgically remove the nucleus from an oocyte.^[^ ^237^ ^]^ In a similar work, the microrobotic arms were assisted with an acoustic levitation system to enable a higher degree of precise 3D manipulation of a single oocyte.^[^ ^238^ ^]^ Spiral‐shaped magnetically powered micromotor were used to transport and release individual fertilized oocytes in a microfluidic chip.^[^ ^239^ ^]^ Such examples demonstrate the potential of micro/nanorobots in assisted fertilization protocols.

### 3.4 Inorganic Agents

**Figure 7:**
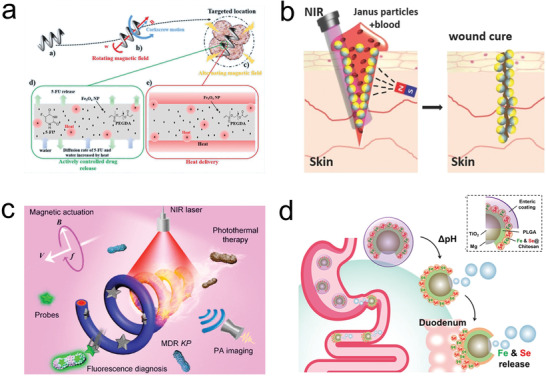
Micro/nanorobot based delivery of inorganic agents. a) Magnetically propelled microrobot for hyperthermia (magnetic heating) therapy. Reproduced with permission.^[^ ^240^ ^]^ Copyright 2019, Wiley. b) Photothermal micromotor based wound healing. Reproduced with permission.^[^ ^243^ ^]^ Copyright 2016, Wiley. c) Magnetically propelled nanorobot for chemo‐photothermal therapy. Reproduced with permission.^[^ ^244^ ^]^ Copyright 2019, American Chemical Society. d) Chemically propelled micromotor for mineral delivery. Reproduced with permission.^[^ ^247^ ^]^ Copyright 2019, American Chemical Society.

Apart from pharmaceuticals, biologics, and cells, inorganic materials offer also hold promise for therapeutic purposes. Their applications include the use of responsive materials that are externally actuated, or the use of metallic ions to treat diseases. The use of sensitive materials that can be activated on‐demand in targeted locations aims to reduce the side effects and dosage requirements of pharmaceuticals while maintaining their therapeutic efficacy. Magnetically propelled microrobots have taken advantage of the capacity of a magnetic material to convert kinetic energy produced by an external magnetic field into thermal energy. The localized heat generated at the target area can be translated in energy to kill cells and have a synergistic effect in combination with medication. Park et al. reported a degradable microrobot capable of hyperthermia, with an increase of the local temperature above 40 °C (**Figure** **7**a). This heat triggered the release of the anticancer drug 5‐fluorouracil and actuating the Fe_3_O_4_ nanoparticles embedded in the robot polymeric matrix.^[^ ^240^ ^]^ Magnetically triggered release of fluorouracil was previously rested using an animal model.^[^ ^241^ ^]^ Moreover, microrobot assisted hyperthermia was recently applied to remove plaques in clogs from a simulated blood vessel. This robot consisted of magnetically coated carbon nanotube that was capable of both chemical and magnetic propulsion.^[^ ^242^ ^]^

Photothermal microrobots were used to seal the superficial wound in a bleeding animal (Figure 7b). The microrobots, consisting of a thermophoretic Janus microstructure with a gold cap, magnetite nanoparticles, and dyes for infrared laser‐assisted tissue welding, were inserted inside an open wound. Upon application of a laser pulse directly into the wound, the micromotor generated a localized temperature increase that denaturalized nearby collagen serving as a “glue” to close the wound. The microrobot was the platform to assist in wound healing, where the pharmaceutical/coagulant was not able to reach the necessary concentrations due to profuse bleeding or lack of localization.^[^ ^243^ ^]^ More recently, magnetic microrobots consisting of magnetized fixed spirulina, helical algae microorganism, demonstrated the targeted chemo‐photothermal therapy triggered by near‐infrared irradiation reaching temperatures above 50 °C (Figure 7c).^[^ ^244^ ^]^ Later studies reported photothermal therapy against a mouse subcutaneous drug‐resistant Klebsiella pneumonia infection model.^[^ ^245^ ^]^

Despite considerable progress in reducing hunger in the world, more than one‐third of the worldwide population still suffers severe nutritional deficiencies, such as anemia commonly caused due to iron deficiency. Thus, there is a need to deliver minerals and ions in the body, as they are hard to absorb due to their low bioavailability and potential adverse side effects.^[^ ^246^ ^]^ The use of magnesium powered microrobots powered by gastric fluid was explored for effective delivery of active minerals, including a cocktail delivery of iron and selenium (Figure 7d). Animal experiments demonstrated the ability of the micromotor to enhance mineral absorption in an anemic mice model. The microrobot‐based therapy resulted in the normalization of diverse, relevant health parameters, including red blood cell count, and hemoglobin.^[^ ^247^ ^]^ In a similar direction, microrobots have also been described as active anion delivery systems. These robots were coated with a polymeric viologen, which can selectively interact with anionic species of different sizes and charge densities. The release of the charge species was achieved by different methods, including electrochemical/photochemical reduction or the presence of an acidic environment.^[^ ^248^ ^]^

Moreover, silver ions have been used in combination with microrobots as bactericidal agents, allowing to deliver silver ions into the bacterial cytoplasm resulting in disruption of their outer membrane without the use of pharmaceuticals.^[^ ^249^ ^]^ Thus, chemically propelled microrobots composed of zeolites have taken advantage of their high absorption capacity to serve as silver ion carriers. The autonomous locomotion of the zeolite microrobot enhanced the interaction between the silver ions and pathogens and increased fluid mixing, resulting in enhanced pathogen killing capabilities.^[^ ^250^ ^]^ Water‐based magnesium micromotors decorated with silver nanoparticles were used toward disinfection and removal of *E. coli* from contaminated samples. The micromotor also contained an inner iron layer that allowed for magnetic recollection and removal of the captured bacteria, leaving the solution without any biological contaminant.^[^ ^251^ ^]^ Magnetically propelled silver‐coated nanocoils were also employed as bacterial killing microrobots by direct contact of the bacteria wall with the active surface of the nanocoil. Efficient decontamination of both Gram‐negative (*E. coli*) and Gram‐positive (*S. aureus*) pathogen stains was demonstrated.^[^ ^252^ ^]^ Nevertheless, the application of microrobots to deliver silver ions has a significant limitation. Most pathogens grow and proliferate inside the mucous wall of the gastrointestinal tract, making a challenge for the silver ions to reach their target. In this direction, an onion inspired multifunctional microrobot was developed as an attracting pathogenic trap for collection and lysis of pathogens (*E. coli*) in solution. The microrobot released a chemoattractant which permitted the dynamic approximation of motile pathogens to the microrobot, and the subsequent release of silver which destroyed the motile pathogen.^[^ ^253^ ^]^

Although scientists have made great demonstrations of micro/nanomotor in targeted delivery, more work on the design aspects toward improving the selectivity and specificity, navigation in biologically relevant environments, controlled cargo release, real‐time‐tracking, and feedback should be conducted for successful in vivo application. All these platforms will foresee a great potential because they could come as a valuable tool for cancer therapy, regenerative medicine, and life extension therapy.

## 4 Surgery

Large‐scale surgical tools do not have analogous micro/nanoscale counterparts, hindering the ability to operate at this small scale and resulting in minimal tissue penetration. Miniaturization of surgical tools could provide distinct advantages due to their small size and ability to access places where catheters and blades cannot. Micro/nanorobotics could serve as surgical tools, aiming to penetrate or retrieve cellular tissues directly. These untethered minimally invasive systems would provide access to regions of the body that their large‐scale robotics counterparts are not able to reach. Besides, they will have the potential to reduce the risk of infection and recovery time.^[^ ^254^ ^]^ Indeed, micro/nanorobot could complement current surgical robotics tools, aiming to increase the precision and control of human surgeons.

### 4.1 Biopsy/Sample Collection

**Figure 8:**
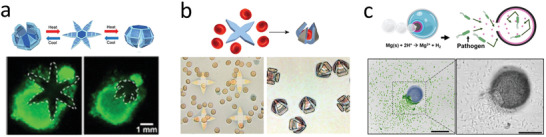
Microrobot based biopsy and sampling. a) Star‐shaped griper collecting tissue. Reproduced with permission.^[^ ^257^ ^]^ Copyright 2015, American Chemical Society. b) Star gripper collecting red blood cells. Reproduced with permission.^[^ ^259^ ^]^ Copyright 2014, Wiley. c) Motile microtrap collecting pathogens. Reproduced with permission.^[^ ^253^ ^]^Copyright 2020, Wiley.

Researchers have illustrated micro/nanorobotic devices that collect tissue samples and bacteria for reduction of damage to tissue, via lowering invasive surgery, and advancing diagnosis. Most untethered microscale robotic devices are still in the centimeter to millimeter range. This size range permits to integrate built‐in communication electronics into the motile robotic pills.^[^ ^255^, ^256^ ^]^ However, further miniaturization from these devices would allow sampling even smaller regions. For instance, star‐shaped grippers that are capable of responding to diverse environmental stimuli to close down and capture tissue have been reported (**Figure** **8**a).^[^ ^257^ ^]^ These tiny medical devices have been evaluated using animal models, demonstrating the ability to excise tissue from a pig bile duct.^[^ ^258^ ^]^ Biocompatible designs made of responsive hydrogels embedded with magnetic alginate microbeads were magnetically guided and presented infrared light‐induced gripping (Figure 8b).^[^ ^259^, ^260^, ^261^, ^262^ ^]^

Micro/nanorobotics were also explored to collect bacteria inside the body. The use of motile robotic collectors could help expand the understanding of the biome. Deployable microtraps were used for sequestering motile bacteria from a liquid environment. The device consisted of microengineered funnels that confine bacteria into subdivision trap chambers.^[^ ^263^ ^]^ More recently, motile microtraps, consisting of an onion inspired multilayer structure, were used to collect motile pathogens. The depletion of the magnesium engine core resulted in a hollow structure that served as a structural trap. Moreover, the inner layer released a chemoattractant (serine) that served to attract nearby motile microorganisms and capture *E. coli* within the microtrap structure (Figure 8c).^[^ ^253^ ^]^ These examples demonstrate the potential of microrobotic devices for biopsy and sample collection. However, the main challenge that these applications face is the ability to preserve the specimen and avoid contamination. Moreover, motile micro/nanorobots could be used to collect diverse biomarkers, such as exosomes^[^ ^264^, ^265^, ^266^ ^]^ (small vesicles excreted from cells) and disease markers.^[^ ^267^, ^268^ ^]^

### 4.2 Tissue Penetration

Robotic systems are useful tools to access deep tissue region that are not reachable trough blood vessel absorption. For example, robotic devices in the centimeter range have been widely used for gastrointestinal assisted delivery, where the robot is used to pierce tissue or collect samples.^[^ ^269^, ^270^ ^]^ The miniaturization of robotic devices could enhance our ability to access even more remote locations inside the body. Most of the literature on tissue penetration is composed of externally powered micro/nanorobots, as external fields are capable of penetrating thick biological tissue. The external energy allows continuous operation in diverse environments and a high degree of control. Magnetic nano/microrobots have been applied to penetrate through the brain of a mouse cadaver. The microdriller robots were inserted intranasally into a mouse brain and propelled under different types of motion, controlled by tuning the applied magnetic field. The rotational motion of the microdrillers presented enhanced penetration when compared to the use of a linear magnetic gradient.^[^ ^271^ ^]^ Moreover, this work was expanded to demonstrate the ability to induce behavioral changes in small mammals by activating magnetic microrobots inserted into neural tissue. The application of a low magnitude external field resulted in an increased level of chewing behavior when compared to control experiments.^[^ ^272^ ^]^ These works illustrate the potential of using mechanical stimulation of neural tissue via unthreaded micro/nanorobots.

**Figure 9:**
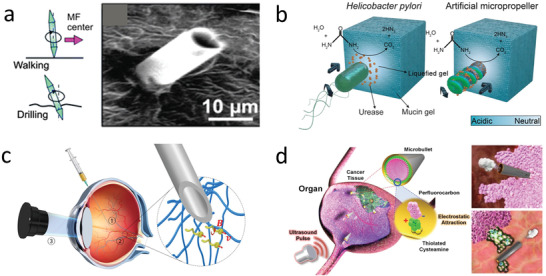
Micro/nanorobots for tissue penetration. a) Magnetic microdriller internalizing into liver tissue. Reproduced with permission.^[^ ^273^ ^]^ Copyright 2013, The Royal Society of Chemistry. b) Magnetic microdriller penetrating mucin gel. Reproduced with permission.^[^ ^274^ ^]^ Copyright 2015, AAAS. c) Magnetic microdriller mobbing inside the eye. Reproduced with permission.^[^ ^277^ ^]^ Copyright 2018, AAAS. d) Ultrasound powered microbullet for tissue penetration and cleaving. Reproduced with permission.^[^ ^91^ ^]^ Copyright 2012, Wiley.

Additionally, rotating microrobot drillers, powered by an external rotational magnetic field, have demonstrated the ability to penetrate deep inside diverse organs. Tubular microdrillers with sharp ends were used to demonstrate proof of surgical applicability (**Figure** **9**b). The microdriller was power by an external magnetic field, enabling to perform different types of motion by modulating the applied frequency, leading to horizontal or vertical orientation, while the structure rotates in axis. The microdriller was forced to penetrate porcine liver tissue while in a vertical position held in place due to the interaction with the rough tissue surface. The microdriller reached a penetration depth of 25 µm after operation for 10 min. The microstructure was recovered using a permanent magnet leaving behind the drilled hole in the liver tissue.^[^ ^273^ ^]^ The resulting hollow cavity could be used to seed cells or implant sustain drug release patches. Enzymatically active biomimetic micropropellers were used for the penetration of mucin gels (Figure 9b). This work aimed to solve the limitation of accessing pathogens located at the mucus layer by using a magnetic microdriller functionalized with the enzyme urease in its outer surface. The external catalytic surface reacted with the mucus layer, locally increasing the pH level, resulting in the liquefication of the mucous, thus allowing the micro/nanorobot to propel through the biopolymeric layer.^[^ ^274^ ^]^

Magnetic powered microrobots have also been used to navigate inside the eye, presenting a high degree of control and biocompatibility. The micro/nanorobots were injected into the vitreous cavity through a surgical opening of the eye. A magnetic coil system was used to navigate the micro/nanorobot through the posterior segment of a rabbit eye.^[^ ^275^ ^]^ The biocompatibility of micro/nanorobot was enhanced by using a polypyrrole coating, presenting minimal inflammatory response in comparison to the not coated counterpart.^[^ ^276^ ^]^ More recently, submicrometer magnetically propelled robots coated in a liquid perfluorocarbon protective layer were used to minimize biofouling and interaction with biopolymeric networks found inside the vitreous body of an eye. The microrobot presented efficient locomotion inside the porcine eye, being able to navigate freely through its structure (Figure 9c). This study presented evidence that robots smaller than 500 nm present unobstructed motion inside the eye as they are smaller in size than the mesh size of the biopolymeric mesh network.^[^ ^277^ ^]^ Microrobots in ocular surgery has shown potential applicability in drug delivery for retinal vein occlusion and surgery for manipulation and peeling of the epiretinal membrane. Another type of small‐scale surgical robotics consists of ultrasound powered microrobots. These devices offer high power “bullet‐like” acoustic droplet vaporization ignition mechanism. The microbullet consisted of a hollow tube (5 µm diameter) filled with perfluorocarbon emulsions electrostatically interacting with the interior surface of the hollow structure. The application of high intensity focused ultrasound pulse directed at the microbullet, vaporized the perfluorocarbon emulsion, rapidly changing its state from liquid to gas, serving as a propellant. Such remarkable speed provided enough thrusts for deep tissue penetration, ablation, and destruction (Figure 9d).^[^ ^91^ ^]^ A modified version of this design results in a functional microscale cannon, where the hollow conical structure was filled with a hydrogel containing 1 µm nanobullets or fluorescent microspheres, and perfluorocarbon emulsion. The application of the focused ultrasound field resulted in the spontaneous vaporization of the perfluorocarbon emulsion, resulting in the rapid ejection of the nanobullets at high speed in the minute of meters per second. The acoustic microcannons were able to deliver nanoparticles into phantom tissue, reaching penetration lengths ≈20 µm.^[^ ^278^ ^]^ Arrays of microcannons have also been translated into transdermal patches, consisting of hundreds of micropores loaded with the therapeutic payload and the perfluorocarbon emulsion. The release kinetics were tested using phantom tissues and pigskin. The use of acoustic droplet vaporization microballistic delivery resulted in enhanced delivery of the anesthetic agent lidocaine when compared to passive diffusion or the use of ultrasound pulses by themself.^[^ ^279^ ^]^ Using a similar firing mechanism solid–gas polymeric nanocups were used for deep tissue penetration. The nanostructure was capable of trapping and stabilizing gas nanobubbles inside its porous structure, which upon exposure to a high focused ultrasound pulse, resulted in the cavitation of the nanobubbles, inducing directional force. The nanocup operational capabilities were tested in a CT‐26 tumor model, presenting enhanced extravasation of IgG model drug and penetration into the vessel wall.^[^ ^280^ ^]^ This phenomenon also produced a detectable cavitation signal that lasts four times longer than other ultrasound contrast agents.^[^ ^281^ ^]^ Moreover, this nancup model was used for deep tissue penetration and delivery of oncolytic viruses.^[^ ^282^ ^]^ When comparing the use of magnetically and ultrasound powered micro/nano robotic surgeons, we should consider their intrinsic advantages. Ultrasound ballistic microrobots can apply higher mechanical force into the tissue, allowing deeper penetration and delivering therapeutic payload independent of the robot microstructure. Nevertheless, they have limited uses and would require an external magnetic field to guide them to their target location. On the other hand, most magnetic actuated robotic microsurgeons have a long‐lasting operation and integrated guidance through the external magnetic field. However, they lack the mechanical force offered by the acoustic droplet vaporization methods.

### 4.3 Intracellular Delivery

More recent developments have aimed at miniaturizing robotic platforms for surgery at the individual cell level.^[^ ^283^, ^284^, ^285^, ^286^ ^]^ The external spatiotemporal control and active manipulation of micro/nanorobots inside living cells have permitted to unprecedented access to the biophysical fundamentals going from gene expression or dynamic mechanical mapping of the intracellular environment to drug delivery and sensing.^[^ ^287^ ^]^ Ultrasound powered microrobots have demonstrated the ability to internalize and propel inside individual living cells. This capability has been used to deliver genetic material inside cells. The use of magnetically powered micromotors has been applied to introduce a higher degree of control inside the cell, with the potential of subcellular surgery. There is potential of microrobotic platforms to be used for novel surgical applications, as they could come as a valuable tool for sample collections, unclogging blocked arteries, and gene delivery.

**Figure 10:**
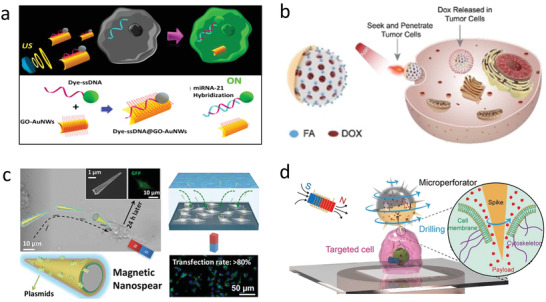
Micro/nanorobot based intracellular internalization. a) Ultrasound microrobot delivery of miRNA. Reproduced with permission.^[^ ^289^ ^]^ Copyright 2015, American Chemical Society. b) NIR powered nanorobot for intracellular delivery. Reproduced with permission.^[^ ^297^ ^]^ Copyright 2020, Elsevier. c) Magnetic microspear delivering plasmids into cell. Reproduced with permission.^[^ ^300^ ^]^ Copyright 2019, American Chemical Society. d) Urchin‐like microperforator for intracellular payload delivery. Reproduced with permission.^[^ ^301^ ^]^ Copyright 2020, Wiley.

The internalization of nanomotors in cells and the manipulation of these artificial machines in the intracellular space was first reported using sound propelled nanomotors inside living HeLa cells.^[^ ^288^ ^]^ Later, the nanorobot internalization inside cancer cells was used for sensing applications or nucleotide, protein, and cargo delivery. As sensing applications inside living cells, they detected miRNA‐21, usually found in cancer cell lines, such as MCF‐7. The miRNA‐21 sensor consisted of a gold nanorobot coated with a fluorescent single‐stranded DNA/graphene oxide and powered by ultrasound fields. The fluorophore in the ssDNA was initially quenched by the graphene oxide, while transported on the nanomotor. However, upon hybridization with the target miRNA, the dyed‐ssDNA probe was displaced from the surface of the nanomachine allowing the fluorescence recovery and miRNA detection (**Figure** **10**a).^[^ ^289^ ^]^ As for cargo delivery, later works applied these gold nanorobot structures for efficient intracellular delivery and application, modified with a rolling circle amplification DNA strand to anchor the siRNA for gene silencing,^[^ ^290^ ^]^ with caspase‐3 for cell apoptosis in acidic environments,^[^ ^291^ ^]^ active intracellular oxygen delivery,^[^ ^292^ ^]^ and with Cas9/ single guide RNA to knockout a GFP reporting gene.^[^ ^293^ ^]^ Using a different and larger structure, nanoshells were internalized and acoustically propelled inside live MCF‐7 cancer cells. The motion was based on acoustic streaming stress over the asymmetric surface of the shell nanomotors. These structures provided higher cargo towing capacity compared to previously described nanowires used for cellular internalization.^[^ ^294^ ^]^ Another design consisted of sharp hollow gold shells that were ultrasound propelled. The microrobot internalization was enhanced by the assistance of a near‐infrared field, inducing penetration by a photomechanical perforation of the cell membrane.^[^ ^295^ ^]^ Stimuli‐responsive intracellular nanorobots were also used to deliver cargo inside Hela cells. These nanorobots consisted of mesoporous silica nanoparticles, including urease‐based chemical engines driven by urea present in the media and pH‐responsive supramolecular nanovalves for cargo‐release. The benzimidazole/cyclodextrin‐urease caps were only opened at low pH delivering the doxorubicin cargo intracellularly at the lysosomal compartments of the Hela cells.^[^ ^296^ ^]^ Mesoporous Janus microrobots were used to thermomechanically percolate inside a cell membrane under NIR irradiation toward releasing doxorubicin (Figure 10b).^[^ ^297^ ^]^ In contrast to ultrasonically or chemically powered techniques that perturb the entire experimental volume, microsurgery using magnetic motors only perturbs the robot and the cell microenvironments.

A plant‐based microrobot capable of dual single‐cell microsurgery along with drug‐was reported. These multitask porous microneedles were composed of biocompatible calcium salts possessing significant fabrication advantages compared to other synthetic cleanroom‐based microdaggers. Coated with an iron–titanium layer, they were driven to cancerous/infected cells, such as HeLa cells under the influence of an external rotating magnetic field. Loaded with the anticancer drug camptothecin, active at acidic pH 5–6, they were capable of selectively release the drug in the partially acidic microenvironment of the tumor. These two functionalities significantly limit the inadvertent side effects on healthy tissue associated with treatment regimens like that of chemotherapy, among others. Further steps improving the adhesion of magnetic coating on the surface on some of the biotubes will still be required. However, the biogenic (plant‐derived) structure assures a biocompatible and robust controlling microsurgery tool with natural cargo‐loading and delivery capabilities.^[^ ^298^ ^]^

A needle‐type microrobot was used for paclitaxel targeted drug intracellular delivery.^[^ ^299^ ^]^ Magnetic nanospears based on gold–nickel–silicon were designed and applied for precision, and targeted intracellular delivery of nucleic acids (Figure 10c). Nanomotors were functionalized via layer‐by‐layer assembly on the external spear layer with plasmids expressing enhanced green fluorescent protein. The magnetic guidance of the nanospears into the U87 glioblastoma cells allowed the penetration into the cell membrane and the intracellular delivery of the plasmid. High rates of plasmid transfection into the cell were achieved and monitored via fluorescence detection, after complete protein expression inside the cell.^[^ ^300^ ^]^ In a similar direction, spiky pollen grain microrobots (urchin‐like) were used for cell drilling. The sharp structure injected doxorubicin into HeLa cells by rolling directly over the cell membrane. The sharp edges detached from the cell as the main structure continued to rotate parallel to the substrate, while the sharp edges drill into the membrane (Figure 10d).^[^ ^301^ ^]^ In another example, titanium‐coated nickel magnetically powered micro/nanorobots were used for transfecting human embryonic kidney cells. The microrobot was loaded with lipoplexes containing plasmid DNA. The outer cationic lipid lipofectamine enabled the efficient transfection of nucleic material into mammalian cells by fusing with the negative membrane and further endocytosis.^[^ ^302^ ^]^ Nevertheless, some of these magnetic material have weak magnetic remanence (iron oxide) or are not biocompatible (nickel). Microhelix composed of iron platinum reported a higher biocompatibility and maintained strong ferromagnetic proprieties. Intracellular transfection of plasmid was used by active targeting of carcinoma by inducing cell expression of green fluorescent protein.^[^ ^303^ ^]^ Magnetic nanorobots have also been used for intracellular surface‐enhanced Raman spectroscopy.^[^ ^304^ ^]^ The development of biocompatible magnetic materials is of great importance to reduce risk and improved performance.

### 4.4 Biofilm Degradation

**Figure 11:**
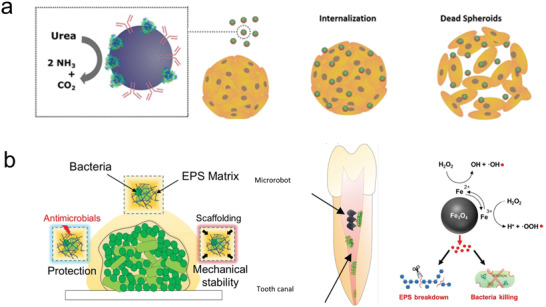
Biofilm degradation. a) Urea‐powered micromotor for degradation of cancer spheroids. Reproduced with permission.^[^ ^309^ ^]^ Copyright 2019, American Chemical Society. b) Magnetic micromotor for catalytic biofilm degradation. Reproduced with permission.^[^ ^310^ ^]^ Copyright 2019, AAAS.

Biofilms and bacterial infections represent a challenge in treatment as diverse pathogens proliferate and colonize different areas of the body resulting in numerous diseases.^[^ ^305^ ^]^ Moreover, biofilms are typically resistant to antibacterial therapeutic, indicating the need of physical methods of treating disease.^[^ ^306^ ^]^ In this direction, different micro/nanorobotic platforms have been employed for mechanically dislodging bacterial pathogens. Magnetic rotating nanowires were utilized to break apart an *Aspergillus fumigatus* biofilm mechanically. The use of rotating nanorobots in combination with an antimicrobial therapeutic agent increased bacterial killing efficacy.^[^ ^307^ ^]^ In other cases, a biohybrid micro/nanorobot, composed of the integration of magnetotactic bacteria (MSR‐1) with mesoporous silica loaded with ciprofloxacin (antibiotic), was explored to apply mechanical stress to *E. coli* biofilm.^[^ ^308^ ^]^ Urease‐powered micro/nanorobot were used for selective targeting, penetration, and treatment of bladder cancer. The micro/nanorobot contained anti‐FGFR3 antibody to selectively bind the outer surface of the 3D cancer spheroids (**Figure** **11**a). Once in there, the ammonia byproduct produced locally enabled the decomposition of the engine, urea, which resulted in a high suppression of spheroid proliferation. A similar strategy could be applied to other biofilms.^[^ ^309^ ^]^ Moreover, catalytic antimicrobial robots were applied to degrade and destroy different models of biofilms. These microrobots consisted of a hydrogel body loaded with iron oxide nanoparticles that serve as the dual function of propulsion via an external magnetic field and as the antibacterial agents. This robotic platform served to swept and remove biofilms over a flat surface, through a blocked capillary tube and to clean biofilms inside an interior tooth model (Figure 11b).^[^ ^310^ ^]^ Hence, microrobots for mechanically destroying biofilms could be expanded to remove blood clots and clean arteries.

## 5 Diagnosis

Motile micro/nanorobots offer unique opportunities for diagnosis, where the microrobot induces an increase in target receptor interaction and fluid mixing. Selective recognition agents of target molecules, including nucleic material, protein, cells, and genetic material, can be employed for analysis and are part of the diagnosis and detection of different biological targets in complex and heterogeneous environments.

### 5.1 Biosensors

**Figure 12:**
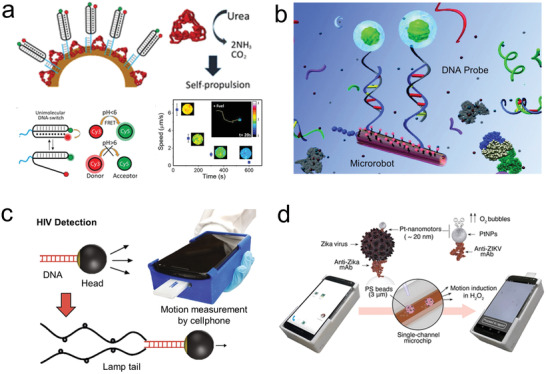
Micro/nanorobot for biosensing. a) Fluorescent sensor for pH monitoring of the surrounding microenvironment using a FRET‐labeled triplex DNA‐based motor. Reproduced with permission.^[^ ^312^ ^]^ Copyright 2019, American Chemical Society. b) Micromotor with oligonucleotide probes for detection of complementary nucleic strains. Reproduced with permission.^[^ ^314^ ^]^ Copyright 2011, American Chemical Society. c) Loop‐mediated isothermal amplification for motion‐based sensing. Reproduced with permission.^[^ ^316^ ^]^ Copyright 2018, Springer Nature. d) Zika virus detection based on motion‐based immunoassay. Reproduced with permission.^[^ ^327^ ^]^ Copyright 2018, American Chemical Society.

Micro/nanorobots have demonstrated capabilities in biosensing to detect diverse biological targets based on changes in motion or fluorescence quenching.^[^ ^311^ ^]^ For example, Sanchez's group reported a Förster resonance energy transfer (FRET)‐labeled triplex DNA pH sensitive nanoswitch for pH‐monitoring of microenvironments (**Figure** **12**a). The DNA sensor detected the ammonia produced as a byproduct of the urea decomposed by the engine of the microrobot.^[^ ^312^ ^]^ A sandwich DNA strand tag with silver nanoparticles was used to detect the Ag^+^ ions induced by the acceleration of a chemically propelled nanorobot. The motion‐driven DNA‐sensing concept relied on measuring changes in the speed of the nanorobot. The concentration‐dependent distance signals were optically visualized. Quantification down to 40 mol DNA and bacterial RNA targets without isolation or purification steps was achieved through the motion of the nanorobot.^[^ ^313^ ^]^

Microrobots functionalized with oligonucleotide probes were applied to detect the complementary nucleic chain of a target DNA or RNA (Figure 12b). Once the target nucleic acid was captured via DNA hybridization, a secondary fluorescent antibody tag provided a signal for quantitative evaluation. The microrobot was capable of isolating samples from different environments including buffer, saliva, and urine.^[^ ^314^ ^]^ Another motion‐based DNA sensing approach was based on catalyze powered microrobot containing single‐stranded DNA capable of capturing a large size particle functionalized with another DNA strand. The microrobot and the cargo oligonucleotides strand are not complementary, but their terminals are complementary to a larger strand that serves as a bridge between them. Thus, when this strain is present in solution the movement of the cargo particle will be detectable through optical microscopy.^[^ ^315^ ^]^

By a similar principle, nanorobots were interfaced with loop‐mediated isothermal amplification of HIV‐1 (Figure 12c). The presence of HIV‐1 RNA strands in solution induced the loop‐mediated isothermal amplification forming a large stem‐looped amplicons that reduced the speed of the nanorobots. This change in acceleration was measured with a 3d printed microscope system attached to the cellphone. This portable platform could detect HIV‐1 virus in patient's samples showing good sensitivity and specificity.^[^ ^316^ ^]^ The DNA sandwich hybridization strategy was expanded based on the functionalization of the nucleic probes inside the engine form a chemically propelled rockets using complementary DNA strands attached to platinum nanoparticles^[^ ^317^ ^]^ and catalase‐loaded DNA strands.^[^ ^318^, ^319^, ^320^ ^]^ The speed of the microrobot was correlated with the concentration of the DNA target. Microrobots were functionalized with aptamer receptors capable of binding, transporting, and isolating thrombin. The experiments demonstrated high selectivity by successfully discriminating against nontarget proteins present in high concentrations. The subsequent release of the protein was achieved by the addition of adenosine triphosphate molecule that binds with the aptamer displacing the captured thrombin. The amount of protein capture was measured quantitatively by the addition of a secondary fluorescence tag.^[^ ^321^ ^]^

Cationic branched polyethyleneimine coated microrobots were used to extract nucleic acid from samples by employing reversible surface charge by changing the environmental pH. The surface charge can absorb the nucleic acid in low pH environment and deabsorbed them in high pH environments, thus presenting a simple method for collection and release of DNA.^[^ ^322^ ^]^ Microrobots functionalized with boronic acid were used for target binding of polysaccharide. The cargo release was achieved by the addition of fructose that had more affinity than the polysaccharide. The surface of the robot had target recognition sites that bind to specific molecules, demonstrated by specific binding of fluorescein isothiocyanate‐labeled avidin.^[^ ^323^ ^]^ Similarly, a Janus based micromotor coated with molecularly imprinted layer was reported for propranolol recognition. The functionalization was achieved via a wax–water pickering emulsion that achieved the generation of propranolol‐imprinted sites by cross‐linking polymerization.^[^ ^324^ ^]^ Multiplexing targeting was also achieved by using nanorobots. This work demonstrated the specific binding of the functionalized gold nanowires with IgG, myoglobin, and thrombin.^[^ ^325^ ^]^ Microrobots functionalized with streptavidin demonstrated to capture biotinylated fluorescent beads. Microfluidic preconcentrating chambers increased the ability of the motor to capture the bead.^[^ ^326^ ^]^

A Zika virus detector was demonstrated based on the motion of a nano/microrobot. This robotic sensor relied on a sandwich assay that employed anti‐Zika mAb coated nanorobots and anti‐Zika mAb coated microbeads. The interaction of these components resulted in the motion of the microbead (Figure 12d). A change in speed of the microbead was detected using a cellphone that indicated different concentrations of the virus. This motion‐based strategy presented a high specificity in the presence of other viruses, including dengue, herpes simplex virus type 1 and human cytomegalovirus.^[^ ^327^ ^]^ Other micro/nanorobots were also sensors for ricin B toxin detection based on an “off‐on” fluorescent mechanism. The microrobot was composed of graphene oxide external layer which can bind to the aptamer tagged with fluorescein‐amidine dye. The target loading due to *π*–*π* interactions, resulted in the quenching of the fluorescent probe. The preferential biding between the ricin and the aptamer results in an increase of fluorescence, thus allowing to measure ricin concentration by optical readout.^[^ ^328^ ^]^ Similarly, reduced graphene‐oxide (rGO) functionalized tubular nanorobots were demonstrated for rapid, cost‐effective, real‐time detection, and effective isolation from mycotoxins (fumonisin B_1_ and ochratoxin A). The operational principle was based on selective recognition of aptamers to target mycotoxins isolation and quantify them based on quenched fluorescence intensity variations. Finally, these rGO functionalized nanomotors showed high sensitivity, selectivity, and suggesting potential applications in biosensing.^[^ ^329^ ^]^ A Janus microrobot functionalized with quantum dots presented the use of “on‐off” fluorescence detection, based in the use of phenylboronic acid which functioned as a recognition receptor for bacterial endotoxins.^[^ ^330^ ^]^ Furthermore, an “on‐off” fluorescent approach was applied for detecting *Clostridium difficile (C. diff)*, which are secreted toxins in a portable and efficient mobile platform. The surface contained functionalized carbon dots that change through fluorescence emission based on the presence of the target toxin. This quantum dot based microrobot protocol was further expanded to isolate *C. diff* toxins discharged from patient's stool. The nanorobot locomotion was controlled by an external magnetic field, which increased the interaction of the toxin with the surface of the nanorobot. The absorption of the toxin over the microrobot surface resulted in the quenching of the quantum dot fluorescence, serving as an optical readout to quantify the presence of *C. diff* toxins in solution (from 0.38 to 17.80 ng mL^−1^).^[^ ^331^ ^]^ Magnetic plasmonic gyro‐nanodisks (GNDs) were used to detect influenza virus (HA1). The working principle was based on the magnetic plasmonic response of the GNDs. These GNDs produced magnetic response and surface plasmon polarization under periodic excitation of an external rotational magnetic field that covert‐induced plasmonic effect into frequencies. Finally, the Fourier‐transform technique was employed to covert increased share stress of GNDs as frequency shift to captured biotarget, such as the influenza virus (HA1).^[^ ^332^ ^]^ Other immunoassays‐based microrobot have been applied to detect carcinoembryonic antigen,^[^ ^333^ ^]^ proteins,^[^ ^334^ ^]^ bacterial toxins,^[^ ^335^ ^]^ cortisol,^[^ ^336^ ^]^ glucose,^[^ ^337^, ^338^, ^339^ ^]^ sepsis,^[^ ^340^ ^]^ and *β*‐galactosidase.^[^ ^341^ ^]^

### 5.2 Isolation

**Figure 13:**
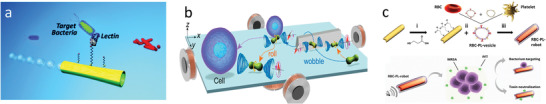
Micro/nanorobot based isolation. a) Microrobot functionalized with antibodies to isolate target bacteria. Reproduced with permission.^[^ ^345^ ^]^ Copyright 2012, American Chemical Society. b) Noncontact manipulation of cancer cells using rotating microrobots. Reproduced with permission.^[^ ^352^ ^]^ Copyright 2018, American Chemical Society. c) Red blood cell/platelet coated nanorobot for synergistic isolation of pathogens and toxins. Reproduced with permission.^[^ ^358^ ^]^ Copyright 2018, AAAS.

Current techniques for isolation and purification of biotargets require long incubation times and multiple washing steps.^[^ ^342^ ^]^ Functionalized micro/nanorobots have been described as “on‐the‐fly” platforms for the rapid isolation of biotargets and infectious pathogens.^[^ ^343^, ^344^ ^]^ For example, nanorobots functionalized with the bioreceptor concanavalin (conA) were used as a tool for real‐time isolation of *E. coli* (**Figure** **13**b). ConA is a mannose‐ and glucose‐binding protein that interacts specifically to the polysaccharid surface of Gram‐negative bacteria. The released of captured bacteria was triggered upon decrease of pH through a glycine‐based dissociation solution.^[^ ^153^, ^345^ ^]^ A similar approach with Anti‐ProtA antibody functionalized microrobots was capable of isolating *S. aureus*, which expresses the complementary protA antibody on its outer membrane. This isolation method presented good selectivity to the target even in excess of yeast cells in solution.^[^ ^346^ ^]^ Orozco et al. reported anti‐*Bacillus globilli* (*B. globilli*) antibody functionalized microrobots to isolate single and multiple *B. globigi spores* in complex biological microenvironment.^[^ ^347^ ^]^

Using a nonspecific isolation approach, *E. coli* was isolated from contaminated water samples based on a “sticky” chitosan hydrogel‐surface coating, which enhanced trapping and killing the pathogens.^[^ ^348^ ^]^ Microrobots functionalized with anti‐carcinoembryonic antigen (anti‐CEA) antibody reported the ability to isolate cancer cells from complex media. The isolation mechanisms were based on specific recognition of the anti‐CEA antibody with the surface antigens overexpressed by pancreatic cancer cells.^[^ ^349^ ^]^ Cancer cells have been also isolated by using physical stimulation. For example, chemical^[^ ^350^ ^]^ and magnetic^[^ ^351^ ^]^ microrobots were able to “pick and drop” cells by pushing them. In another case, peanut shape colloid reported the noncontact fluidic manipulation and transport of cells based on the generation of localized microstreaming flows capable of trapping the cell (Figure 13b). This principle was explored to isolate and transport multiple cells inside microfluidic holes.^[^ ^352^ ^]^ Similar nonmanipulation of living cells has also been achieved using acoustic streaming.^[^ ^353^, ^354^ ^]^

Blood cell‐based coating has also been described for the rapid isolation of pathogens and toxins. Ultrasound^[^ ^355^ ^]^ and chemically^[^ ^356^ ^]^ powered nanorobots coated with red blood cell membranes have been demonstrated as motile sponges for isolating toxin in biological environments. This approach served as a decoy to absorb toxins that would commonly bind and kill red blood cells. Moreover, magnetic helical nanorobots coated with plasma membrane of human platelet, which showed platelet‐mimicking properties, including platelet‐adhering pathogens, such as *S. aureus*.^[^ ^357^ ^]^ The integration of a hybrid cellular membrane coating on the surface of the nanorobots resulted in a synergistic isolation of both pathogen and toxin (Figure 13c). The platelet membrane isolated the pathogen *S. aureus*, while a red blood cell membrane absorbed the *α*‐toxin absorption secreted from the captured pathogens.^[^ ^358^ ^]^

### 5.3 Physical Sensor

**Figure 14:**
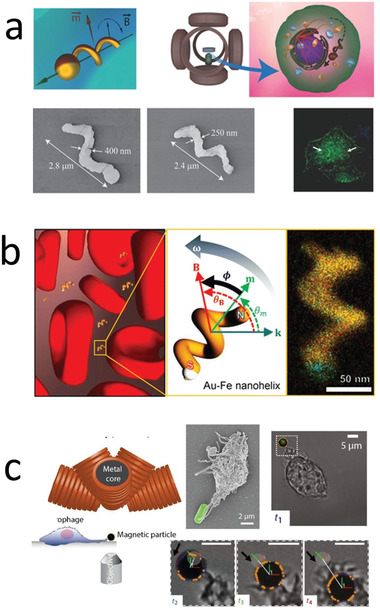
Micro/nanorobots as mechanical probes. a) Nanorobot of measuring intracellular mechanical properties. Reproduced with permission.^[^ ^359^ ^]^ Copyright 2018, Wiley. b) Nanorobot for biofluid rheology. Reproduced with permission.^[^ ^364^ ^]^ Copyright 2016, American Chemical Society. c) Nanorobot for measuring mechanical forces of a macrophage. Reproduced with permission.^[^ ^369^ ^]^ Copyright 2017, AAAS.

Micro/nanorobots have potential as label‐free biomechanical probes. For example, helical nanorobots driven by rotating magnetic fields were used to probe the mechanical properties inside living cells (**Figure** **14**a). This nanomotor faced the challenge of low adherence to their surrounding environment inside the cells, which in certain cases, can lead to reducing mechanical response. However, motion of helical magnetic nanorobots was successfully explored in Hela, kidney, and endothelial cells environments after incubation and internalization.^[^ ^359^ ^]^ This robotic intracellular probe approach was also demonstrated to measure viscosity,^[^ ^360^ ^]^ and adhesive forces inside metastatic cancer cells.^[^ ^361^ ^]^ Piezoelectric microrobots were also reported as wireless probes for neural stimulation mapping and differentiation of single cell using the piezoelectric effect produced by an external ultrasound field.^[^ ^362^ ^]^ For example, electrically powered microrobot were used as motile electrodes capable of deforming cells, in which the resulting distortion of the cell nucleus correlated with detectable dielectrophoretic potential wells.^[^ ^363^ ^]^

Viscosity is an important parameter for medical applications. Nevertheless, the measurement of rheological properties in biological fluids is challenging as they contain a mixture of multiple biocomponents. Rotating nanorobots coils as mechanical sensors were also studied for determining the rheological properties of a microscopic volume (Figure 14b). The rotating nanorobot probed the viscosity of the solution by measuring the lag phase between nanorobot orientation and the external oscillating magnetic field. Thereby, determining the torque applied to the particle by the medium. Because the nanorobots were smaller than the suspended red blood cells, they could selectively probe only the fluid phase.^[^ ^364^ ^]^ Chemically powered microrobots were also explored as viscometers. The velocity of the microrobot allowed to estimate the fluid viscosity of a solution.^[^ ^365^ ^]^ Similarly, different physical parameters (pressure and flow rate) were obtained based on the propulsion of a microrobot.^[^ ^366^ ^]^

The study of microphages hunting behavior and immune host defense at the micro‐environment presents a challenge due to the lack of physical probes that can interact with phagocytes in a similar fashion that pathogens.^[^ ^367^, ^368^ ^]^ In this direction, magnetic microrobots with 5‐degrees of freedom were studied to mimic the predator–prey interactions, hunting, and phagocytes behavior in macroscopic biological organisms, particularly in the immune host defense at microscale environment (Figure 14c). Dynamic translational resistance or rotational forces were also applied as a magnetic torque to arbitrarily position microrobots as prey near to the microphages, and measure the exert forces, and rotation torques as optical displacements.^[^ ^369^ ^]^

## 6 Medical Imaging

The transition from in vitro to in vivo research has addressed the need to integrate microrobots with medical imaging platforms.^[^ ^370^, ^371^, ^372^ ^]^ A key aspect for medical micro/nanorobotic translation in the clinic will rely on individual or population monitoring, with consideration of tissue background signal.^[^ ^373^ ^]^ In this direction, micro/nanorobots could benefit the current medical imaging modalities, as they could be easily localized and guided inside the body, and even send signals to induce triggered release. Current conventional medical imaging techniques study organ and tissue physiology, and more recently, track the distribution of molecular imaging agents and nanoparticles inside a patient's body. The ability to monitor and guide individuals and groups of nanomotors inside the body will be essential to achieve their widespread application. Therefore, the unique advantages of each imaging platform and processing methods should be considered, as each imaging modality is better suited for different organ compositions and depth‐of‐focus inside the body.^[^ ^374^ ^]^ The main challenge, regarding untethered micro/nanorobotic research, is the ability to distinguish the micro/nanorobot structure from their environment (background subtraction) with sufficient acquisition resolution to track each step of motion in a 3D environment. Due to the complexity of the data acquired, algorithms, instead of medical staff would be required to identify and follow the microrobot motion through the body. In this direction, we need to consider how to tag each micromotor by incorporating contrast imaging materials or distinct engineered shapes that allow us to limit the effect of background noise. The imaging modalities used in combination with micro/nanorobots include optical, magnetic, acoustic, and radionuclide imaging.

### 6.1 Optical Imaging

**Figure 15:**
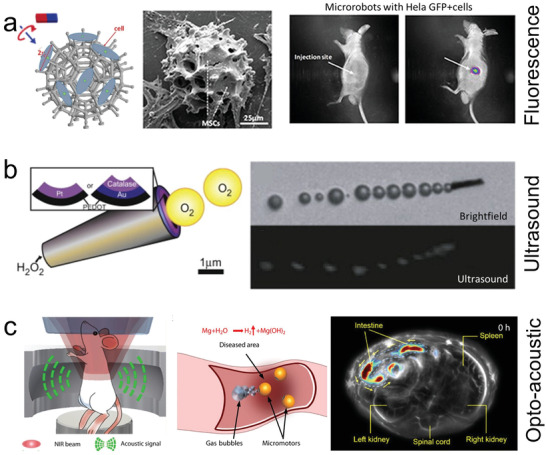
Medical imaging of microrobots. a) In vivo fluorescence imaging of magnetically driven microrobot carrying cells into a mouse flank. Reproduced with permission.^[^ ^229^ ^]^ Copyright 2018, AAAS. b) In vivo photoacoustic imaging of a magnesium propelled motor located at a mouse intestine. Reproduced with permission.^[^ ^382^ ^]^ Copyright 2013, Elsevier. c) In vitro ultrasound imaging detecting bubbles generated by a chemically propelled microrobot. Reproduced with permission.^[^ ^382^ ^]^ Copyright 2019, AAAS.

The diagnosis of the disease remains a challenge for clinicians and researchers, as most cases are asymptomatic until advanced stage due to lack of sensitivity and specificity to accurately detect premalignant lesions. In this case, micro/nanorobots can enhance current imaging capabilities. Initial works in micro/nanorobots employed optical methods, which included catheter cameras and light radiation, which offer a robust imaging capability at a relatively low cost. Thus, optical cameras have shown the position of micromotors inside a rabbit eye,^[^ ^275^, ^375^ ^]^ and with an endoscopic camera a microgripper inside a pig bile has been visualized.^[^ ^258^ ^]^ Although handy, these ingestible cameras have limited access inside the body, can cause patient discomfort and, in some cases, are not able to detect nano/micrometer size structures. In this direction, noninvasive fluorescence imaging could help achieve a high‐resolution quantification and localization of microrobots. In vivo fluorescence of organic molecules or inorganic fluorescent nanoparticles was monitored with a charge‐coupled device camera which captured the light emitted from an animal body^[^ ^229^ ^]^ (**Figure** **15**a). The fluorescent signal was further overlaid over the actual picture of the animal allowing a spatial localization of the molecular imaging agent. Thus, the widespread application of imaging microrobots inside the body is easily achievable by modifying the surface of microrobots with fluorescent molecular imaging agents.

Fluorescence imaging techniques have thus typically studied to visualize the position of micromotors in living animals. Swarms of magnetically actuated helices functionalized with near‐infrared probes (NIR‐797) where monitored inside the peritoneal cavity of a mouse using whole‐body fluorescence imaging.^[^ ^376^ ^]^ Spirulina microalgae coated with superparamagnetic magnetite (Fe_3_O_4_) nanoparticles was applied to track a swarm of micromotors located inside the subcutaneous tissue and the intraperitoneal cavity of nude mice using fluorescence‐based in vivo imaging.^[^ ^377^ ^]^ Other of the multiple examples of biohybrid microrobot as carriers of imaging agents is one of the first examples of micromotor imaging which consisted of a bacteria biohybrid microrobot loaded with 40 nm nanoparticles that contained the luciferase gene. These micromotors were injected intraperitoneally into a mouse for the delivery of a luciferase DNA plasmid inside nearby cells and expressed the luciferase protein, which resulted in the luminescence in different organs.^[^ ^378^ ^]^ Similarly, the migration of biohybrid microrobot bacteria loaded with Cy5.5‐coated polystyrene microbeads was imaged inside a tumor site by the signal produced by fluorescent dye.^[^ ^379^ ^]^ Moreover, microrobots carrying living cells have been imaged using fluorescence emitted from the cell.^[^ ^229^, ^230^ ^]^ In these cases, the fluorescent imaging capabilities where essential to illustrate the successful and target delivery by microrobots into its desired location.

There is still room for improvement in optical imaging for micro/nanorobots. Thus, recent works have aimed to distinguish individual micro/nanorobots, taking advantage of Janus microrobot asymmetric “reflective” properties. In this case, the use of a customized optical IR imaging setup coupled with an image correlation software was employed to monitor single unlabeled microrobots in real‐time underneath phantom and ex vivo mouse skull tissues. The change in reflectivity of micromotors enable their tracking through phantom thicknesses.^[^ ^380^ ^]^

### 6.2 Ultrasound Imaging

Ultrasound imaging is another candidate that offers a biocompatible cost‐effective alternative to visualize micro/nanorobots in real‐time. The interaction of the ultrasound pulse with tissue containing different reflection properties produced a distinct echo that is recorded and transformed into an image. Although ultrasound external field is widely developed to power micro/nanorobots, the employment of ultrasound as an imaging modality remains limited.^[^ ^381^ ^]^ In vitro studies have illustrated the ability to detect the position of a micro/nanorobot that propels through solutions by catalyzing hydrogen peroxide into a trail of oxygen microbubbles (Figure 15c).^[^ ^382^ ^]^ The main limitation of this system is that the imaging system is detecting the microbubbles responsible for propulsion, but not the micromotors themselves. Therefore, the obtained data points would have to be processed to provide an accurate position of the micro/nanorobot. The main challenge of ultrasound relies on its limited resolution and limited selection of contrast agents, consisting mostly of stabilized microbubbles. To solve this limitation, the optoacoustic imaging system offers the resolution and penetration depth of ultrasound combined with the specificity of optical methods. Photoacoustic imaging working principle consists on the emission of optical pulses that are absorbed by tissues, resulting in a thermoelastic expansion that generates ultrasound waves. A transducer is then able to collect the information and transform it into images of the molecular imaging agents inside tissues. A recent study illustrated the ability to visualize micromotors coated with molecular imaging agents, inside an ex vivo chicken breast model, with high contrast and specificity.^[^ ^380^ ^]^ In another case, the ability to localize micro/nanorobots with chemically propelled engines inside a living mouse was achieved using photoacoustic imaging (Figure 15b). Additional near‐infrared light irradiation is also able to activate the propulsion of the microrobot by disintegrating a protective layer.^[^ ^383^ ^]^ More recently, magnetotactic biohybrids coated with a polydopamine layer, which enhanced the photoacoustic detection signal and subsequent photothermal therapy were also reported. This study served a proof of concept for the treatments of pathogens in an in vivo model.^[^ ^245^ ^]^

### 6.3 Magnetic Imaging

**Figure 16:**
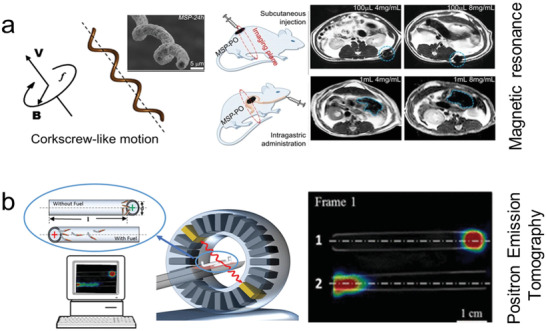
Other medical imaging applications. a) Magnetic resonance imaging of helical micromotors for intragastric region Reproduced with permission.^[^ ^377^ ^]^ Copyright 2017, AAAS. b) Radionucleotide based imaging of microrocket coated with iodine isotope. Reproduced with permission.^[^ ^386^ ^]^ Copyright 2018, American Chemical Society.

Magnetic imaging is one of the most robust methods to image micro/nanorobotic structures inside the body. Magnetic imaging resonance (MRI) employs magnetic fields to visualize biological tissue with a high degree of spatial resolution and contrast. The imaging mechanism is based on the absorption and re‐emission of electromagnetic radiation or hydrogen nuclei under the presence of a strong magnetic field. The energy produced during this event is collected and process to give insight into different physical and chemical information about the molecules at the imaging locations. Different contrast can be generated by fine‐tuning the magnetic field pulses. In particular, when compared to optical imaging, magnetic imaging has higher resolution and penetration depth. Moreover, it also reduces the undesired side effect of ionizing radiation of X‐ray imaging. In the context of micro/nanorobotic research, magnetic materials embedded in the micro/nanorobot structure can generate distortion in the incident magnetic field, producing a signal in the resulting image. The magnetic susceptibility of the materials allows them to be distinguished from nearby tissue. In recent years, MRI imaging has been applied to visualize magnetic structures inside small mammals. Recent works have used magnetic helical microswimmers made from Spirulina microalgae coated with superparamagnetic magnetite (Fe_3_O_4_) nanoparticles.^[^ ^377^ ^]^ In this case, the magnetic coating serves as the engine to convert the external magnetic field into motion and as an imaging contrast, eliminating the need for any further surface modification. In vivo experiments with these robots demonstrated the ability to track a swarm of these microstructures inside a mouse stomach (**Figure** **16**a).

MRI has also shown applicability for wirelessly guiding microswimmer's through the body^[^ ^384^ ^]^ and as a therapeutic tool via magnetic hypothermia.^[^ ^385^ ^]^ Nevertheless, these works include different fields for imaging and guiding. Therefore, the operation of both types of fields at the same time is a challenge, reducing the ability to perform operations in real‐time. Alternatively, when both fields are applied at the same time, there could be a delay between microrobot actuation and image processing.

### 6.4 Radionuclide Imaging

Radionuclide imaging technologies are also another powerful tool in medical imaging. They have unique advantages, as they offer molecular information and sensitivity. Proton emission tomography (PET), is based on emitting positrons that can break down radionuclides, thus, generating gamma rays that a scanner can detect and use for mapping the studied region. Different nuclides can be employed to target specific organs or biological processes. Although the radiation offers deep tissue penetration, it could result in adverse health events in prolong uses, thus limiting the available time to image. A PET system was used to track a large group of Au/poly(3,4‐ethylenedioxythiophene/Pt chemically propelled microrobots coated with iodine isotope (Figure 16b).^[^ ^386^ ^]^ Soft thermoresponsive magnetic microrobots were used for emission computed tomography imaging.^[^ ^387^ ^]^ X‐rays have also shown limited examples for microrobotics.^[^ ^388^ ^]^ X‐ray radiation has been demonstrated to power Janus microparticles,^[^ ^389^ ^]^ and to detect star‐shaped microgrippers inside the gastrointestinal tract.^[^ ^258^ ^]^ As a general overview of the medical imaging section, each method has its unique advantage. Optical and ultrasound imaging have a relatively low‐cost, although inferior penetration depth when compared to magnetic and radionucleotide imaging.

## 7 Outlook

In summary, the use of microrobots in precision medicine has shown a diverse plethora of applications in different fields including the delivery of pharmaceuticals, biologics, genes, and living cells; surgical tools for biopsy, tissue penetration, intracellular delivery, or biofilm degradation; diagnostic tools including physical and chemical biosensors or isolation tools; and optical, ultrasound, magnetic, and radionuclide imaging tools (**Table** **1**). The most developed application is target delivery and current efforts are mainly focused on animal testing. However, imaging is required in combination with delivery, surgery, or diagnosis to understand the dynamics of the micro/nanorobots and leverage their efficiency and capabilities. Current research of nano/microtools suggests the constant narrowing of the gap between precision medicine and micro/nanorobotics. Nevertheless, each application faces different challenges toward potential clinical translation. For delivery and surgery applications, micro/nanorobots would require operating in hard to access regions of the body, therefore recovery/degradation strategies are of great significance to ensure they would not pose a risk to the patient health. Diagnostic tests are often conducted in vitro, therefore the main challenge for microrobots as diagnostic tools would rely on improving their scalability and enabling high‐throughput detection. The main challenge in medical imaging applications is the ability of each imaging modality to distinguish in real time individual and large groups of micro/nanorobots forming a background tissue. This challenge would require high frame rate acquisition at high magnification, thus producing large quantities of data. The use of machine learning algorithms could enable to rapidly evaluate the data forclose loop operation of microrobots in vivo.

**Table 1:** Overview of current trends of micro/nanorobotics in Precision Medicine, including delivery, surgery, diagnosis, and medical imaging

Application	Description/justification	Examples	Challenges
Delivery 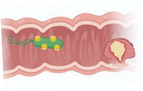	Use of micro/nanorobots to deliver target payload with higher spatiotemporal resolution versus passive diffusion	Pharmaceuticals Biologics Living cells Inorganic therapeutics	Dosing Selective release Biodegradation‐retrieval
Surgery 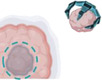	Use of micro/nanorobots as surgical tools to access remote locations, while aiming to reduce invasive surgical procedures	Biopsy/sampling Tissue penetration Intracellular delivery Biofilm degradation	Force generation Biodegradation‐retrieval Sample recovery
Diagnosis 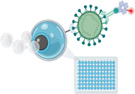	Use of micro/nanorobots to enhance fluid mixing and mechanical force to increase sensing capabilities	Biosensor Isolation Physical sensor^−^	High throughput Scalability Cost
Imaging 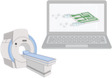	Provide feedback loop mechanism to enable other applications and exploration of the body	Optical Ultrasound Magnetic Radionuclide	Higher resolution Higher frame rate Data processing

Moreover, micro/nanorobots face safety, technical, regulatory, financial, and market challenges to be overcome before they are translated to clinical uses. We will itemize those in detail below. Although there is a long road ahead, the use of microrobots in precision medicine has the potential to improve diagnosis and treatment, which could lead to improving a patient's life. Microrobots could help in precision medicine and reduce the cost and discomfort associated with major surgical procedures.

### 7.1 Safety and Biocompatibility

Ensuring the safety and biocompatibility of medical micro/nanorobots is a critical prerequisite for their widespread implementation in the clinic.^[^ ^390^ ^]^ Ideally, the micro/nanorobot will perform its intended function by either delivering a cargo, performing surgery, or providing a diagnostic signal. Then, after completing its task, it would be reabsorbed in the body or retrieved by a medical device or by excretion. The degradation of the micro/nanorobot can be tuned for a specific biological environment or by time‐dependent materials proprieties of the robotic device.^[^ ^391^, ^392^, ^393^ ^]^ On the other hand, catheters like devices would retrieve the micro/nanorobot.^[^ ^394^, ^395^ ^]^ The material composition of the micro/nanomotor will determine how long their structure could last inside the body. For example, most magnesium‐based micromotor engines are fully depleted, while PLGA‐based magnetic microengines could last for days. Moreover, the introduction of foreign materials could produce an inflammatory response by the patient's immune system.^[^ ^396^ ^]^ Therefore, designing micro/nanorobots with biocompatible coatings with tunable surface charge, stretchability, hydrophilicity, and morphology; could limit adverse results and expand their longevity. Micro/nanorobots are not currently used in clinical trials, but initial progress has been made in preclinical animal tests reporting hematology and blood chemistry with minimal adverse effects.^[^ ^177^, ^377^ ^]^ Ethical considerations should be kept in mind when designing micro/nanorobot animal experiments, as these initial tests increase the minimal risk for the animal without direct benefit. Moreover, the results obtained by animal studies do not always translate to humans. Nevertheless, they have the potential to yield generalizable knowledge with translational benefit to a general patient population. No micro/nanorobotic technology covered in this review has been tested in humans. Therefore, there is still much work to be done to evaluate the long terms side effects that micro/nanorobots could have on human health.

### 7.2 Technical and Regulatory Challenges

**Figure 17:**
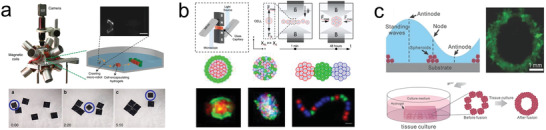
Biofabrication of microrobots and cellular microstructures. a) Hydrogel robot fabricated by magnetic assembly. Reproduced with permission.^[^ ^225^ ^]^ Copyright 2014, Springer Nature. b) Tunable fabrication of cellular microstructures using magnetic levitation. Reproduced with permission.^[^ ^402^ ^]^ Copyright 2018, Wiley. c) Bioacoustic fabrication of organoids. Reproduced with permission.^[^ ^409^ ^]^ Copyright 2015, Wiley.

In the realm of technical challenges, mass fabrication of the microrobotic system presents a problem to solve. Over the next decade, with the development of sophisticated bio fabrication manufacturing techniques and continuous innovations on biocompatible materials, micro/nanorobots could become safe to use in clinical applications.^[^ ^397^, ^398^ ^]^ In this direction, researchers should focus on the use of novel high throughput fabrication methods that assists in the interfacing of tissue engineering and nanoengineering. Such development fulfills the needs of functionality and biocompatibility required by medical devices.^[^ ^399^ ^]^ These individual parts could be assembled into more sophisticated modular structures. For example, our group has reported the use of magnetic assembly methodologies to build untethered magnetic micro/nanorobots powered by magnetic fields. This type of hydrogels‐based robot was composed of multiple building blocks to create a cooperative assemble of parts capable of interacting with their environment and performing predetermined tasks. The microrobot was fabricated by incorporating magnetic nanoparticles inside the hydrogel (**Figure** **17**a).^[^ ^225^ ^]^ An external magnetic field was used to guide the self‐assembly of the magnetic hydrogels into patterned 2D and 3D modular structures with different designs and material proprieties, such as mass density, porosity, and elastic modulus.^[^ ^400^, ^401^ ^]^ Another type of magnetic assembly based on magnetic levitation was used to self‐assemble living cells into complex configurations embedded into a 3D extracellular matrix (Figure 17b).^[^ ^402^ ^]^ The use of magnetic levitation is based on the use of two magnets with the same poles with a microfluidic channel embedded between them. The cells are resuspended in a cellular medium containing dissolved gadolinium‐based paramagnetic agents that, in conjunction with the magnetic field is capable of inducing force to levitate living cells based on the difference between the magnetic susceptibility of the medium and cells.^[^ ^403^ ^]^ Magnetic levitation has unique advantages including the ability to use different types of cells with no requirements for magnetic nanoparticles that would need to be removed later from the constructs.^[^ ^404^ ^]^ Recently, magnetic levitation has been used to guide the bioassembly of 3D tissue constructs in space marking a seminal advance in 3D bioprinting and biofabrication.^[^ ^405^ ^]^ Another promising biocompatible technique consists on the use of bioacoustic‐enabled fabrication for assembling a large number of microscale units (synthetic beads, cells, spheroids) into reconfigurable and ordered symmetric structures. The large‐scale patterns can be modulated by fine‐tuning the applied acoustic field. The bioacoustic fabrication method has unique advantages, as it eliminates the requirement of supportive materials and can preconcentrate a large number of cells in a few seconds.^[^ ^406^ ^]^ Moreover, we have demonstrated the use of bioacoustic fabrication with types of cellular designs, including for cardiac tissue,^[^ ^407^ ^]^ brain‐like constructs,^[^ ^408^ ^]^ ring‐shaped cellular robots,^[^ ^226^ ^]^ and organoids (Figure 17c).^[^ ^409^ ^]^ The development of biorobots has the potential to create fully autonomous micro/nanorobots in the interface of growth and assembly. Moreover, integrating biomaterials into the robot design could increase its safety and cloak it from a patient immune system.

**Figure 18:**
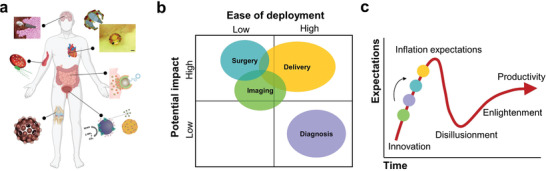
Micro/nanorobots’ clinical translation outlook. a) Operation of micro/nanorobots in diverse regions of the body. b) Characterization of medical micro/nanorobots based on their potential and ease of deployment. c) Developmental trends of emerging technologies.

Micro/nanorobots have been tested in diverse regions of the body including the gastrointestinal tract, blood vessels, brain, and knee cartilage (**Figure** **18**a). Nevertheless, a challenge moving forward relies on demonstrating their safety. There are multiple regulatory bodies, such as FDA agency in the United States, China Food and Drug Administration in China, European Medicines Agency (EMA) in Europe, Japan Pharmaceutical Manufacturers Association (JPMA) in Japan, among others that will evaluate and approve the use of micro/nanorobotic platforms in the clinic. To pass such regulatory hurdles, new technologies need to demonstrate their safety and efficacy.^[^ ^410^, ^411^ ^]^ The probability of getting approval is historically very low and is also very costly and time‐consuming. Most regulatory agencies worldwide classify medical devices based on their potential risk to a patient's well‐being. As an example, Class I is considered a low or minimum risk. Deciding which kind of purpose the micro/nanorobot will be used for, will determine their classification. Thus, affecting the time required for clinical translation. Some of the sensing applications that are performed in an external assay could be classified as class I, if their output provides a qualitative response rather than a quantitative result. A micro/nanorobot would be class II or III if they partially or fully penetrate a patient either through a body opening or surface. Although it might seem easier to bypass the regulatory system by choosing a lower risk application with faster ease of deployment, such as micro/nanorobotic based diagnostic platforms, a higher risk/high potential application, such as delivery or surgery could provide a competitive advantage to the micro/nanorobot in the long run, as illustrated in the prioritization table on the basis of potential and ease of deployment shown in Figure 18b. Researchers should consider outsourcing some of the clinical validation work required for FDA approval to a private research contract organization.^[^ ^412^ ^]^ Artificial intelligence and machine learning could also help to increase the safety of micro/nanorobots. Local path planning algorithms could help train micro/nanorobots to navigate in the unknown and dynamically changing biological environments, thus avoiding hitting obstacles and getting stuck inside the body.^[^ ^413^ ^]^

### 7.3 Financial and Market Challenges

When analyzing the technology readiness of micro/nanorobots as an emerging technology, their clinical translation potential is still in the early stages.^[^ ^414^, ^415^, ^416^, ^417^ ^]^ The typical technology maturity development of emerging technologies as develop by Gartner is shown in Figure 18c. The maturity of the field will progress based on its commercialization outlook. Therefore, one of the major barriers to their clinical translation involves securing the cost of funding early developments in university and private research. Nondilute funding is available but is quite competitive. Therefore, novel technologies could benefit from academic‐industry partnerships. The main financial cost that should be considered involves scientific staff, cost of equipment and materials, regulatory costs, and intellectual property. Universities offer a unique setting for developing technological innovation as multiple fields of study (engineering, medicine, business, law) are under the same institution. Researchers in the field should expand their patenting, as technological developments without proper intellectual property protection are unlikely to secure funding from the private sector. Commercial enterprises are also essential to advance the field of robotics, as profits could be reinvested into research and development of new micro/nanorobotic technology.

Finally, there is the market risk. Although micro/nanorobots have shown promising results in in vitro and in vivo studies, they currently lack proof of concept application that offers a distinct potential advantage over the state of the art, for example, increased therapeutic efficiency, reduced side effects, discomfort, or lower cost. In order to achieve lab to market transition, commercial enterprises in micro/nanorobotics should identify unmet needs that could validate the market of medical microrobots. Initial micro/nanorobotic commercial ventures should focus on complementing existing medical devices. For example, by integrating them with catheters to reach reduced and inaccessible regions of the body. Although there is a long road ahead, once micro/nanomotors have initial proof of concept in human subjects, leveraging micro/nanorobots in precision medicine will improve diagnosis and treatments, which could lead to improve patient's life.

## Conflict of Interest

Prof. Utkan Demirci (U.D.) is a founder of and has an equity interest in: i) DxNow Inc. ii) Koek Biotech, iii) Levitas Inc. iv) Hillel Inc. U.D.'s interests were viewed and managed in accordance with the conflict of interest policies.
